# Regulation of *KLRC* and *Ceacam* gene expression by miR-141 supports cell proliferation and metastasis in cervical cancer cells

**DOI:** 10.1186/s12885-024-12794-6

**Published:** 2024-09-03

**Authors:** Emad Dabous, Mai Alalem, Ahmed M. Awad, Khaled A. Elawdan, Ahmed M. Tabl, Shorouk Elsaka, Walid Said, Adel A. Guirgis, Hany Khalil

**Affiliations:** 1 Department of Molecular Biology, Genetic Engineering and Biotechnology Research Institute, University of Sadat City, P.O Box 79, Sadat City, Egypt; 2 Microbiology and Chemistry Department, Faculty of Science, Benha University, Benha, Egypt

**Keywords:** MiR-141, Cervical cancer, Cell proliferation, KLRC genes, Ceacam genes, Metastasis

## Abstract

**Introduction:**

MicroRNAs (miRNAs) are single RNA molecules that act as global regulators of gene expression in mammalian cells and thus constitute attractive targets in treating cancer. Here we aimed to investigate the possible involvement of miRNA-141 (miR-141) in cervical cancer and to identify its potential targets in cervical cancer cell lines.

**Methods:**

The level of miR-141 in HeLa and C-33A cells has been assessed using the quantitative real-time PCR (qRT-PCR). A new miR-141 construct has been performed in a CMV promoter vector tagged with GFP. Using microarray analysis, we identified the potentially regulated genes by miR-141 in transfected HeLa cells. The protein profile of killer-like receptor C1 (KLRC1), KLRC3, carcinoembryonic antigen‐related cell adhesion molecule 3 (CAM3), and CAM6 was investigated in HeLa cells transfected with either an inhibitor, antagonist miR-141, or miR-141 overexpression vector using immunoblotting and flow cytometry assay. Finally, ELISA assay has been used to monitor the produced cytokines from transfected HeLa cells.

**Results:**

The expression of miR-141 significantly increased in HeLa and C-33A cells compared to the normal cervical HCK1T cell line. Transfection of HeLa cells with an inhibitor, antagonist miR-141, showed a potent effect on cancer cell viability, unlike the transfection of miR-141 overexpression vector. The microarray data of HeLa cells overexpressed miR-141 provided a hundred of downregulated genes, including KLRC1, KLRC3, CAM3, and CAM6. KLRC1 and KLRC3 expression profiles markedly depleted in HeLa cells transfected with miR-141 overexpression accompanied by decreasing interleukin 8 (IL-8), indicating the role of miR-141 in avoiding programmed cells death in HeLa cells. Likewise, CAM3 and CAM6 expression reduced markedly in miR-141 transduced cells accompanied by an increasing level of transforming growth factor beta (TGF-β), indicating the impact of miR-141 in cancer cell migration. The IntaRNA program and *miRWalk* were used to check the direct interaction and potential binding sites between miR-141 and identified genes. Based on this, the seeding regions of each potential target was cloned upstream of the luciferase reporter gene in the pGL3 control vector. Interestingly, the luciferase activities of constructed vectors were significantly decreased in HeLa cells pre-transfected with miR-141 overexpression vector, while increasing enormously in cells pre-transfected with miR-141 specific inhibitor.

**Conclusion:**

Together, these data uncover an efficient miR-141-based mechanism that supports cervical cancer progression and identifies miR-141 as a credible therapeutic target.

**Supplementary Information:**

The online version contains supplementary material available at 10.1186/s12885-024-12794-6.

## Introduction

Cervical cancer is a significant public health problem and the fourth most common cancer among women worldwide, with about 569,847 new cases and 311,365 deaths registered annually. By 2030, there is an estimation expected that the number of cervical cancer cases will increase by around 50% worldwide [1]. In recent decades**,** the morbidity and mortality of cervical cancer have reduced in many countries due to the widely implemented prevention programs and the effective screening programs (Pap smear) provided in many countries [2]. Nevertheless, in low and middle-income countries, the screening programs have focused only on offering the test to women in primary health care centers more than in health clinics. Therefore, the current prevention programs in developing countries are not significantly decrease the morbidity and mortality of cervical cancer [3]. Typically, cancer cells go through different stages in which cell factors are implicated in each step. For instance, the overexpression of epidermal growth factor receptor (EGFR), mutant proteins KRAS, BRAF, and depletion in tumor suppressor effectors such as killer-like receptor C (KLRC), P53, and PTEN have been associated with cancer initiation and development [4, 5]. Meanwhile, vascular endothelial growth factor (VEGF), tumor necrosis factor-alpha (TNF-α), and epidermal growth factor (EGF) are classified as angiogenic activators [6]. Notably, cell adhesion proteins (Ceacam family) and hepatocyte growth factor (HGF/SF) are the critical modulators of cancer progression, particularly during metastasis [7].

Importantly, microRNA (miRNA) is a small non-coding RNA molecule that can post-transcriptionally regulate gene expression of particular mRNA [8, 9]. Cellular miRNAs play a crucial role in a broad spectrum of biological progress, including cell division, cell death, evaluation, hematopoiesis, and the pattern of nervous system [10]. Hundreds of miRNA non-coding genes were recognized and identified in mammalians and many phylogenetically conserved species [11]. The non-coding genes lin-14 and lin-41, which encode to miRNAs lin-4 and let-7, were firstly connected with loss-of-function that leads to defects in developmental timing in nematodes [12]. In mammalian, miRNAs mediated gene expression includes different interactions, either near perfect or partial complementation associated with cellular protein complex, namely the RNA-induced silencing complex (RISC). These interactions lead to RNA degradation and/or translation inhibition of targeted genes [13].

Accumulated evidence has shown the utility of miRNAs in cervical cancer initiation, metastasis, and drug resistance [14]. For instance, miR-126 has been implicated in cervical cancer diagnosis, while supplementation of miR-143 or inhibition of miR-21 has been shown to incorporate into cervical cancer therapy [15]. As an onco-miRNA, miR-21 is overexpressed in cervical cancer and implicated in the expression of the tumor suppressor gene *programmed cell death 4 *(*PDCD4*) and the chemokine *CCL20* involved in tumor differentiation and metastasis [16, 17]. Likewise, miR-19a and miR-19b are upregulated in cervical cancer cells and incorporated in cell proliferation and cytopoiesis of malignant-type HeLa and C33A cells [18]. In this way, the miR-200 family, including miR-141, has been reported as an essential miRNA aberrantly expressed in several human cancer and involved in cell proliferation, migration progression, and cancer invasion and diagnosis [19]. In addition, a recent study suggested the increasing level of miR-141 in cervical squamous cell carcinoma and its involvement in the carcinogenesis of cervical cancer through the regulation of the tumor suppressor gene *PTEN* [20]. These suggestions indicate the possible role of miR-141 in cervical cancer development. Based on this, we deeply investigated the molecular interaction behind the upregulation of miR-141 in cervical cancer cells. We identified the regulated genes by miR-141, which validates its role in cancer cell proliferation and cancer metastasis.

## Material and methods

### Cell lines

All cell lines were obtained from (VACSERA, Giza, Egypt) and regularly checked for mycoplasma contamination. Cell lines, including human cervical cancer HeLa cell line, C-33A cell line, and the normal cervical keratinocytes cell, HCK1T cell line, were cultured in Roswell Park Memorial Institute (RPMI) 1640 media supplemented with 25 mM HEPS, 4 mM L-glutamine, and 10% of heat-inactivated bovine serum albumin (BSA) and were incubated at 37 °C and relative humidity of 95% [21, 22].

### Cloning strategy

To perform the miR-141-over-expression vector, GFP plasmid with CMV promoter was used to clone the full-length miR-141 between the CMV promoter and GFP sequence. To generate the construct pCMV-miR-141-GFP, the full-length miR-141 was isolated from HeLa cells using the following specific oligonucleotides containing restriction sites specific for Sac1 and Pst1: Sac1-For-5`-gagctccgctaacactgtctggtaaag -3`and Pst1-Rev-5’-5`-ctgcaggtgcagggtccgaggt -3`. Accordingly, total RNA was extracted from cultured HeLa cells using TRIzol (Invitrogen, USA). The extracted total RNA was then purified using the RNeasy Mini Kit (Qiagen, USA), followed by the purification of small miRNAs using the RNeasy MinElute Cleanup Kit (Qiagen, USA) according to the manufacturer’s protocol. cRNA was synthesized from total RNA using miR-141-Pst1-Reverse primer and the following reagents: 4 µl 10X RT buffer (Promega), 4 µl RNA (1 µg/µl), 4 µl dNTPs (25 mM), 0.5 µl RNase inhibitor (5 U/µl) (Promega) and 0.5 µl M-MuLV reverse transcriptase (100 U/µl) (Promega) up to 20 µl final volume using RNase free water. The mixture was incubated for 3 h at 42 °C followed by 10 min at 95 °C to stop the reaction. The synthesized cRNA was then used to amplify the full-length miR-141 using conventional PCR by the indicated miR-141 specific primers. PCR product was then loaded in agarose gel 1%, eluted and digested with Sac1 and Pst1 as well as pCMV-GFP plasmid as recommended in NEB conditions. The open vector was loaded in agarose gel, eluted and incubated overnight at 4 °C with miR-141 digested fragments and 5U of T4 DNA ligation, 4 µl 5X ligation buffer (Promega), 2 µl from cRNA (100 mM), 2 µl from the vector (1 µM) adjusted to a final volume of 20 µl. The performed constructs were checked for the successful one with the right orientation of miR-141 sequence. To validate the direct interaction between miR-141 and its identified targets, the individual coding sequence of KLR1, KLR3, CAM3, and CAM6, by which miR-141 could interact, was cloned upstream of the luciferase reporter gene in the pGL3 vector with SV40 promoter (Promega, Germany). First, we amplified the specific fragment from genomic DNA of cultured HeLa cells using Platinum SuperFi DNA polymerase (Invitrogen, Germany) based on the predicted seeding region of miR-141 and targeted mRNA using the following primer sequences;

- KLRC1-(880)-for (5'- AGTTTGCTGGCCTGTACTTCGAAGAAC-3') and
- KLRC1-(1141)-rev (5'- AGGTGTGTTGTAAATTGTATTAAATTA -3'),
- KLRC3-(600)-for (5'- ACAATAAATGGTTTGGCTTTCAAACAT-3') and
- KLRC3-(700)-rev (5'- TGATATAAGTCCACGTACATGTAGCA-3'),
- CAM3-(450)-for (5'- ACCCTACAAGTCATAAAGTCAGATCTT-3') and
- CAM3-(550)-rev (5'- TGGGGTTGGAGTTGTTGCTGGAGATGG -3'),
- CAM6-(500)-for (5'- ATGAAGAAGCAACCGGACAGTTCCATG-3') and
- CAM6-(600)-rev (5'- TTCTGAACCTCAGGTTCACAGGTGAAG-3').

The pGL3-control vector was digested with HindIII using the FastDigest HindIII (Thermo Scientific, USA). The digested vector was loaded and electrophoresed in 1% agarose gel and was then eluted from the gel using the QIAquick Gel Extraction kit (Qiagen, USA). The blunt-end protocol was used to insert the amplified fragment in the pGL3 open vector using a TOPO-Blunt-End Cloning kit (Invitrogen, Germany). Briefly, the amplified fragment was incubated with the pGL3 opened vector in a concentration ratio of 3:1 and five units of T4 DNA ligase (Invitrogen, Germany) for 30 min at room temperature. The miR-141 overexpression vector and luciferase reporter constructs were transformed into the competent *E. coli* strain by heat shock (42ºC for 45 s). The transformed *E.coli* was grown in a Petri dish with agar media containing ampicillin at 37 °C for 3 days. A selected single colony of *E.coli* was grown in the broth media at 37 °C overnight. Maxi-Prep kit (Qiagen, USA) was used to amplify the constructs, which were then checked for the correct insert and proper orientation by restriction digestion using the restriction sites map of the new construct prepared by the cloning manager program.

### Transfection protocol

HeLa cells were cultured in 6-well plates at 10 × 10^3^ cells per well and incubated overnight. Cells with confluency of 70% were transfected with 1.25 µg/ml pCMV-GFP-miR-141 construct using Lipofectamine LTX (Invitrogen, USA), according to the manufacturer’s protocol. Transfected cells were incubated for two days, followed by total RNA isolation for qRT-PCR, staining for flow cytometry, or protein purification for immunoblotting assay. Other cells seeded in 6-well plates with the same confluency were transfected with a respective inhibitor antagonist, miR-141 (5-′ACAACCACTGTCTGGTAAAG-3′) [23]. According to the manufacturer’s instructions, the cells were transfected with 1.25 µg of inhibitor/ml using 20 µl Lipofectamine LTX (Invitrogen, USA), prepared in 500 µl optimum media. Cells transfected with the same concentration of transfection reagents were considered as control-transfected cells. Transfected cells were incubated for two days. The knockdown efficiency of miR-141 and the relative expression of indicated genes were monitored in transfected cells using qRT-PCR. Immunoblotting and flow cytometry assays for KLRC1, KLRC3, CAM3, and CAM6 protein were assessed on day two post-transfection [24]. For cotransfection with luciferase reporter constructs, HeLa cells were cultured in black 96/well plates with a 10 × 10^3^ cell/well density and incubated overnight. Then, the cells were transfected with either miR-141 overexpression vector or miR-141 specific inhibitor using 125 ng from each, 5 µl Lipofectamine LTX, and 20 µl optimum media to transfect each well. Two days later, the transfected cells were cotransfected with 125 ng of pGL3 constructs using 5 µl Lipofectamine and 20 µl optimum media per well. The cells were then incubated for 24 h and prepared for luciferase dual assy.

### Transfection cytotoxicity and proliferation assay

Transfected cells' cytotoxicity and viability were monitored to achieve the anti-proliferation properties of miR-141 overexpressing vector. Accordingly, HeLa cells were seeded in a 6-well plate in triplicates and were transfected with miR-141 overexpressing vector or specific inhibitor as previously described. Forty-eight hours post-transfection, cell morphology and number of living cells were monitored using an inverted microscope and hemocytometer, respectively [25, 26]. To investigate cell viability upon transfection, HeLa cells were cultured in triplicate in 96-well plates with a density of 10 × 10^3^ cells per well, followed by transfection with different concentrations of miR-141 overexpressing vector or specific inhibitor (1,25–20 µg/ml). Other cells treated with the same concentration of transfection reagents served as control-transfected cells. Cell viability rate was achieved using an MTT colorimetric assay kit (Sigma-Aldrich, Germany). Briefly, transfected cells were washed using phosphate buffer saline (PBS), and 100 µl complete media was added to each well. 10 µl MTT solution was added to each well with a gentile piptting. The plate was then incubated for 1 h at 37ºC. Finally, 100 µl SDS-HCl solution was added to each well in the plate and was incubated for 4 h at 37ºC. Cell viability was calculated according to the amount of converted water-soluble MTT to an insoluble formazan which was determined by optical density at 570 nm.

### Annexin-V assay

The early and late apoptosis detection in transfected cells was performed using an annexin-V (FITC)/propidium iodide (PI) assay kit (BD Biosciences). In brief, 10 × 10^4^ HeLa cells were cultured in a 25 ml cell culture flask and incubated overnight under the same pre-described conditions of cell culture. The cells were then transfected with 1.25 µg/ml of either miR-141 overexpression vector or specific inhibitor and incubated for 24 h. Transfected cells were then collected and washed twice with PBS, and were resuspended in the kit's Binding Buffer. Then, 100 μl of the cell suspension was incubated with 5 μl of Annexin‐ V conjugated fluorescein isothiocyanate (FITC) and 5 μl of PI for 15 min in the dark at room temperature. Then 500 μl of the binding buffer was appended, and the cells were analyzed by flow cytometry [27].

### Microarray analysis

Total RNA was purified from transfected cells using TRIzol and RNA preparation method (Invitrogen, USA) using glycogen as a carrier following the manufacturer’s protocol with a few modifications. Xylene and ethanol treatment in the precipitation step were excluded. To reduce RNA degradation, the incubation steps at 55 °C and 80 °C were shortened to 12 instead of 15 min. RNA was eluted from the column with RNase-free water, quantified by NanoDrop, and stored at -80 °C. RNA quality was confirmed by an Agilent 2100 bioanalyzer (Agilent Technologies) and a NanoDrop 1000 spectrophotometer. In brief, 600 nanograms of total RNA were reverse transcribed and amplified using an oligo-dT-T7-promotor primer, and the resulting cDNA was labeled either with Cyanine 3-CTP or Cyanine 5-CTP. The microarray experiments were scanned using a DNA microarray laser scanner (DNA Microarray Scanner BA, Agilent Technologies) at 5 µm resolution using Ambion ship and Exiqon ship according to the manufacturer’s instructions. The original microarray images were analyzed with the Image Analysis/Feature Extraction software G2567AA (Version A.7.5, Agilent Technologies) using default or standard miRNA microarray settings. Non-uniformity outlier flagging was performed with a 5 × default value of the constant coefficient of variation for features ((CV)2 Term (A) = 0.55) and background ((CV)2 Term (A) = 0.45). Intensities were normalized by local background correction and rank consistency filtering with Lowess-normalization. The intensity ratios were calculated using the most conservative estimate of error between the Universal Error Model and propagated error [28]. A Two-fold change expression cut-off for ratio experiments was applied with anti-correlation of color-swapped ratio profiles, depicting the microarray analysis as highly significant (*P *value < 0.01).

### Monitoring miR-141 expression level

The relative expression level of miR-141 was achieved in transfected cells using qRT-PCR. Accordingly, total RNA was extracted from transfected HeLa cells (48 h post transfection) using TRIzol (Invitrogen, USA). The isolated total RNA was then purified using the RNeasy Mini Kit (Qiagen, USA), followed by the purification of small miRNAs using the RNeasy MinElute Cleanup Kit (Qiagen, USA) according to the manufacturer’s protocol. RT-PCR was used to detect the relative expression of miR-141 in two different steps. First, cDNA was performed from small miRNAs by using reverse transcriptase reaction followed by the amplification step using miR-141 and RNU6B specific TaqMan microRNA assays (Applied Biosystem, Darmstadt, Germany), according to the manufacturer’s protocol. Levels of RNU6B were used for normalization. To perform cDNA from small miR-141, the following reagents were prepared for reaction: 0.15 µl dNTPs (100 mM), 1 µl reverse transcriptase (50 U/µl), 1.5 µl 10X reverse transcriptase buffer, 0.2 µl RNase inhibitor (20 U/µl), 5 µl purified miRNAs (10 ng/µl) and 1 µl from each primer up to final volume of 20 µl using RNase free water. The mixture was then incubated for 30 min at 42 °C followed by 5 min at 85 °C to inactivate the enzyme. The resulting cDNA then was used as a template to amplify both miR-141 and RNU6B by using the following parameters in the quantitative real-time PCR (qRT-PCR) machine: 95 °C for 5 min, 40 cycles (95 °C for 15 s, 60 °C for 15 s and 72 °C for 15 s) and 72 °C for 3 min.

### Gene expression profiling

The relative gene expression of indicated genes was monitored using qRT-PCR., In addition, qRT-PCR was used to perform cDNA construction and amplification in one step using the purified total RNA as a template. Total RNA from transfected HeLa cells was extracted 48 h post-transfection using TRIzol and purified using the RNeasy Mini Kit (Qiagen, USA). The relative expression of *KLRC1, KLRC3, CAM3*, and *CAM6* was detected using the QuantiTect SYBR Green PCR Kit (Qiagen, USA) in miR-141 transduced HeLa cells or miR-141 depleted cells. The oligonucleotides in Table 1 have been used as specific primers for indicated genes. Level of amplified GAPDH was used for normalization. The following reagents were prepared for each reaction; 10 µl SYBR green, 0.2 µl RNase inhibitor (20 U/µl), 0.25 µl reverse transcriptase (50 U/µl), 1 µl purified total RNA (100 ng/µl) and 0.5 µl from each primer up to a final volume of 20 µl using RNase free water. According to the manufacturer’s protocol, the following PCR parameters were sued to construct and amplify cDNA, in one step, from a total RNA template: 50 °C for 30 min, 95 °C for 3 min, 40 cycles (95 °C for 30 s, 60 °C for 15 s, 72 °C for 30 s) and 72 °C for 10 min [29].

**Table 1:** Oligonucleotides sequences used for quantifying miR-141 and mRNA of indicated genes

Description	Primer sequences 5'-3'
**MiR-141-sense**	CGCTAACACTGTCTGGTAAAG
**MiR-141 antisense**	GTGCAGGGTCCGAGGT
**MiR-U6-sense**	GCTTCGGCAGCACATATACTAAAAT
**MiR-U6-antisense**	CGCTTCACGAATTTGCGTGTCAT
**KLRC1-sense**	ATATGTCTCCCAGGAAGTCTCTGT
**KLRC1-antisense**	GGCCTGCTATAGCAACAGTGATT
**KLRC3-sense**	AAGGTTTACTGCCACCTCCAGAA
**KLRC3-antisense**	TTACCAATGTAATAACAACTGTT
**CAM3-sense**	TCATAGTCACTGATAATGCTCTACC
**CAM3-antisense**	GGAGACTGAGGGTTTGTGCT
**CAM6-sense**	TCAGCCACTGGCCTCAATAG
**CAM6-antisense**	TCTGGTCCAATCTGCCAGTC
**GAPDH-sense**	TGGCATTGTGGAAGGGCTCA
**GAPDH-antisense**	TGGATGCAGGGATGATGTTCT

### Flow cytometry assay

Flow cytometry was used to achieve the kinetic expression of KLRC1, KLRC3, CAM3, and CAM6 in transfected HeLa cells. Accordingly, the transfected cells were washed twice with PBS and then were trypsinized for 3 min. The complete RPMI medium was added to the trypsinized cells, then the cells were centrifuged for 5 min at 3000 rpm. The supernatant was discarded, and the pellet was resuspended in PBS for washing and incubated for 3 min in cold methanol for fixation. The cells were resuspended in PBS with Triton-X-100 (0.1%) and incubated for 3 min for permeabilization. For primary antibody staining, the cells were resuspended and incubated overnight at 4 °C in the PBS supplemented with 1% BSA and the diluted mouse polyclonal anti-CAM3 (1–500) (Sino Biological, China). After washing with pure PBS, the cells were centrifuged and resuspended in the PBS with 1% BSA and 1–1000 secondary antibody (goat anti-mouse IgG, Alexa Fluor 488, Abcam, USA). The cells were then incubated in dark conditions for 2 h. The same instructions were followed for staining the cells with the primary and secondary antibodies against CAM6 using rabbit monoclonal anti-CAM6 (1–500) (Ab 275,022, Abcam, USA) and goat anti-rabbit IgG (1–1000) (Alexa Fluor 594, Abcam, USA) [30]. For staining KLRC1 and KLRC3, the primary antibodies; rabbit polyclonal anti-KLRC1 (Sigma-Aldrich, USA) and mouse monoclonal anti-KLRC3 (Sigma-Aldrich, USA) were used. The same secondary antibodies; goat anti-rabbit (Alexa Fluor 488, Abcam) and goat anti-mouse (Alexa Fluor 594) have been used to achieve the kinetic expression of KLRC1 and KLRC3, respectively. Finally, the flow cytometry assay (BD Accuri 6 Plus) was used to assess the protein levels using a resuspended pellet in 500 µl PBS [31, 32].

### Immunoblotting analysis

The protein expression of KLRC1, KLRC3, CAM3, and CAM6 was double-checked using an immunoblotting assay. Total protein was extracted from transfected HeLa cells using the complete lysis and extraction buffer, RIPA (ThermoFisher, USA). Then the protein was denaturized using a loading buffer containing 10% sodium dodecyl sulfate (SDS) and boiling at 95–100 °C for 5 min. 100 ng of denaturized protein was loaded in 10% sodium dodecyl sulfate–polyacrylamide gel, and the electrophoresis was carried out for 4 h at 75 V using the Bio-Rad Mini-Protean II electrophoresis unit. The protein bands were then transferred onto nitrocellulose membranes (Millipore, MA, USA) using the Bio-Rad electro-blotting system (Bio-Rad Mini Trans-Blot Electrophoretic Transfer Cell). For blocking, the membrane was incubated for one hour at room temperature in 30 ml of Tris Buffered Saline containing 5% non-fat dry milk and 0.1% Tween-20 (pH 7.5). Then the membrane was incubated overnight at 4 °C with either mouse monoclonal anti-CAM3 (1–1000) or rabbit monoclonal anti-CAM6 (1–1000) diluted in the blocking buffer. For detecting KLRC1 and KLRC3 protein expression, the membrane was incubated for 2 h at room temperature (RT) with rabbit polyclonal anti-KLRC1 (1–500) or mouse monoclonal anti-KLRC3 (1–500) diluted in the blocking buffer. The membrane was then washed twice using the WesternBreeze solution 16x (Invitrogen, USA) followed by 2 h incubation at room temperature with mouse monoclonal anti- β-actin (Sigma, Hamburg, Germany). Finally, the membranes were washed twice and incubated for 2 h at RT with either anti-moue or anti-rabbit ready-to-use 2° Solution Alkaline-Phosphates (AP) Conjugated (Invitrogen, USA). After washing, the membranes were cut carefully just below the protein marker band 40 kDa to reach the β-actin at 42 kDa and both KLRC1 and KLRC3 at 27 kDa. The individual membranes were stained to detect the expression band of CAM3 and CAM6 at 30 and 40 kDa, respectively. The chromogenic detection of expected bands was performed immediately using AP substrate (WesternBreeze, Invitrogen, USA).

### ELISA and luminometer assays

ELISA assay was used to measure the released interleukins, IL-4, IL-8, IL-10 m and TGF-β using homospians ELISA kits (Abcam 100,750, Abcam 100,575, Abcam 185,986, and Abcam 100,647, respectively). HeLa cells cultured in 96-well plates were overnight incubated. Then the cells were transfected with either miR-141 overexpression vector or miR-141 specific inhibitor (1.2 µg/ml) followed by an incubation period of (0, 4, 8, 16, 24, and 48 h). At each time point, the cells were lysed using 1X cell lysis buffer (Invitrogen, USA). Then, 100 µl of the lysed cells were transferred into the ELISA plate reader and incubated for 2 h R.T. with 100 µl control solution and 50 µl 1X biotinylated antibody. Then 100 µl of 1X streptavidin-HRP solution was added to each well of samples and incubated for 30 min in the dark. 100 µl of the chromogen TMB substrate solution was added to each well of samples and incubated for 15 min at R.T., away from the light. Finally, a 100 µl stop solution was added to each well of samples to stop the reaction. The absorbance of each well was measured at 450 nm [33]. Luciferase activities were determined 24 h post transfection with pGL3 constructs using the Firefly Luciferase Assay, Dual Luciferase Assay (Sigma-Aldrich, Germany). In brief, the transfected cells were lysed by adding 10 µl lysis buffer (Promega, Germany) to each well with carefully pipetting up/down. 10 µl firefly solution was added to each well with gentle pipetting. The luciferase activity indicated by light production was measured immediately on the luminescence microplate readers (SpectraMax Luminometer*,* USA).

### Prediction tools and data analysis

The Freiburg RNA online tool; IntaRNA program was used to predict the possible interactions between miR-141 and targeted sequences of each gene. The miR-141 sequence was obtained from mirbase website, while individual gene sequence has been obtained from National Library of Medicine (https://www.ncbi.nlm.nih.gov). The i*n-silico* online tool, *miRWalk*, was also used to validate and confirm the direct interaction between miR-141 and identified genes using the following link http://mirwalk.umm.uni-heidelberg.de. For quantification the cycle threshold (Ct) of each investigated gene expression, delta-delta-Ct equations were used as previously described: (1) delta-Ct = Ct value for gene—Ct value for GAPDH, (2) (delta–delta-Ct) = delta-Ct for experimental–delta-Ct for control, (3) relative expression of targeted gene = ( 2^−delta−delta ct^) [34, 35]. Statistical analysis was done using the student’s t-test between two groups. *P-*value ≤ 0.05 was considered statistically significant.

## Results

### Antagonist miR-141 effectively regulates HeLa cell proliferation

**Fig. 1:**
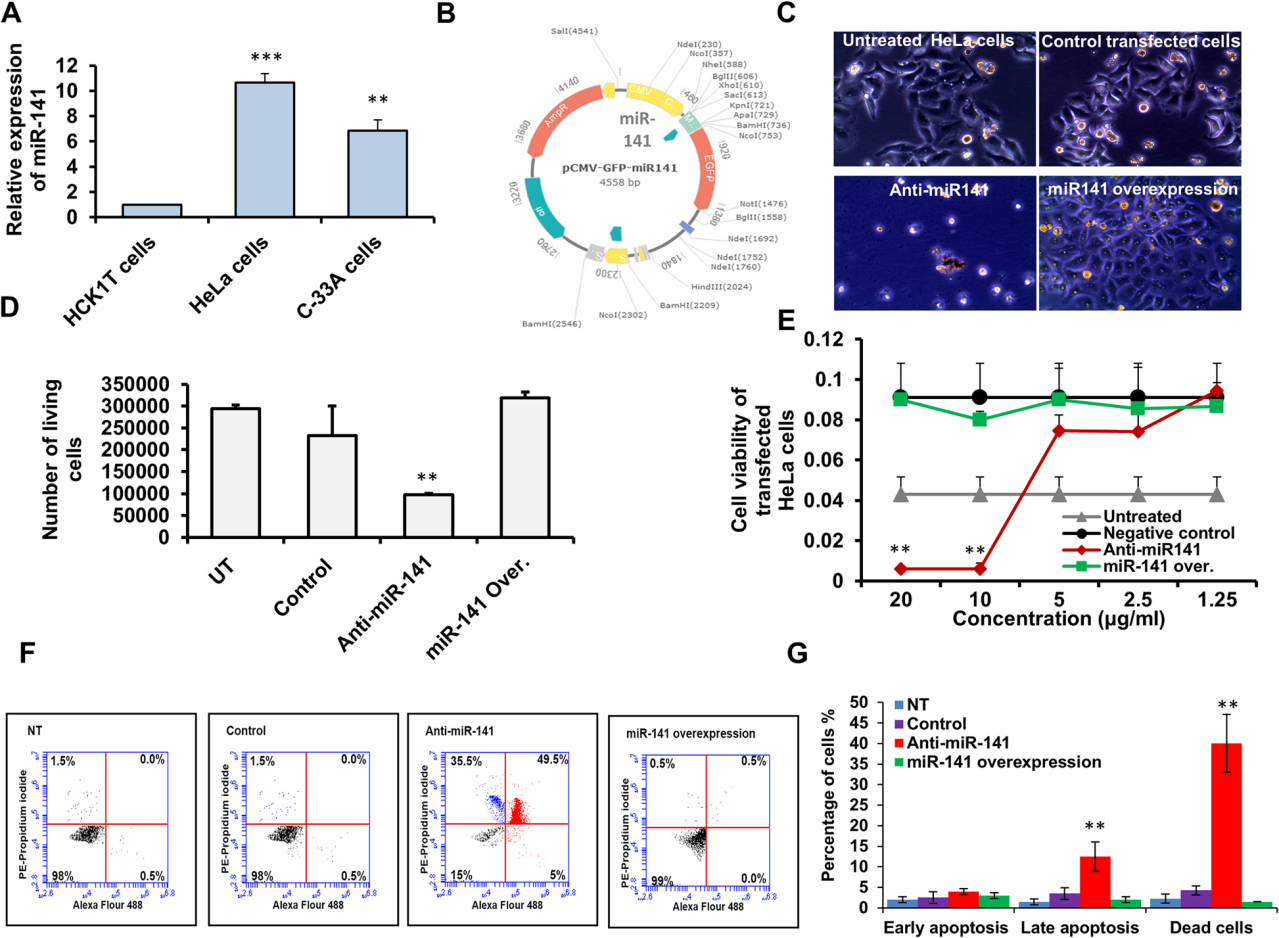
Correlation of miR-141 in cervical cancer cell proliferation. **A** Quantification of miR-141 expression level in cervical cancer HeLa and C-33A cells compared with the normal cervical HCK1T cells indicated by fold changes using the qRT-PCR. **B** Schematic representation of pCMV-miR-141-GFP construct map showed the cloned full-length miR-141 downstream of CMV promoter cassette using online Molbiotools. **C** HeLa cell morphology indicated by inverted microscope upon 48 h of transfection with either miR-141 overexpression vector or an inhibitor antagonist miR-141 compared with control-transfected and untreated cells. **D** After transfection with the miR-141 inhibitor or the overexpression vector, the number of living HeLa cells. **E** Cell viability rate of transfected HeLa cells with different concentrations of the miR-141 inhibitor or the overexpression vector indicated by the absorbance rate of treated cells with MTT agent. **F** HeLa cells were transfected with either the miR-141 overexpression vector or its inhibitor for 48 h, and then cells were stained with (Annexin V + /Propidium *Iodide* (PI)). The early and late apoptotic singling and dead cells were monitored using flow cytometry. **G** The percentage of transfected cells with positive signals for early or late apoptosis and the percentage of dead cells indicated by flow cytometric assay. Error panels indicate the standard deviation (STD) of three independent experiments. Student two-tailed *t*-test used for statistical analysis, (*) indicates *P-values* ≤ 0.05, (**) indicates *P* ≤ 0.01, and (***) indicates *P* ≤ 0.001

The expression level of miR-141 was first achieved in different cervical cancer cells, including HeLa cells and C-33A cells, compared with its expression level in the normal cervical cells HCK1T cells. Interestingly, as shown in Fig. 1A and Table 2, the relative expression of miR-141 significantly increased in both cervical cancer cell lines reached a tenfold change in HeLa cells compared with the normal HCK1T cells. To determine whether miR-141 plays any role in HeLa cell proliferation, we constructed a miR-141 overexpression vector using a pCMV-GFP vector with a CMV promoter and the original enhanced GFP sequences. The whole sequence of miR-141 was inserted downstream of the CMV promoter region and upstream of the GFP original sequences (Fig. 1B). The new construct pCMV-GFP-miR-141 was then used to transfect HeLa cells compared with those transfected with the specific miR-141 inhibitor. Interestingly, the transfected cells with the miR-141 overexpression vector showed accelerated growth rates and large-scale cell congestion, evidenced by cell morphology presented in Fig. 1C and compared to cells transfected with the miR-141 inhibitor. In contrast, the cell morphology of HeLa cells transfected with the miR-141 inhibitor showed marked obstruction in the growth of cells compared with control transfected cells and large areas devoid of any development of cancer cells (Fig. 1C). Furthermore, the number of living cells significantly reduced in HeLa cells transfected with the inhibitor antagonist miR-141 while showing nonsignificant differences compared with control-transfected cells and untreated cells (Fig. 1D and Table 3). Likewise, the cell viability rate of HeLa cells transfected with the miR-141 inhibitor strongly decreased in dose-dependent inhibitor concentrations (Fig. 1E). In contradiction, cells transfected with the miR-141 overexpression construct showed neglected differences in cell viability rate compared with control-transfected cells, while revealed an increasing viability rate compared to untreated cells (Fig. 1E). To confirm the influence of miR-141 in the PCD of transfected cells, we assessed the early and late apoptosis in addition to the percentage of dead cells upon transfection using the Annexin V. As presented in Fig. 1F, our findings revealed that the lower signaling of apoptosis and the lower percentage of dead cells were achieved in non-treated (NT) cells, control transfected cells, and cells transfected with miR-141 overexpression vector. In contrast, the transfection with the miR-141 inhibitor showed increasing levels of dead cells in almost 40% of stained cells and increasing levels of the late apoptotic signaling detected in approximately 50% of stained cells (Fig. 1F and G). These data demonstrate the potential role of miR-141 in regulating HeLa cell proliferation and PCD.

**Table 2:** Quantification analysis of miR-141 in cervical cancer cell lines

Conditions	Expression fold changes	STD	*P values*
**HCK1T cells**	1	00.00	
**HeLa cells**	10.3**	0.72	0.002
**C-33A cells**	6.23**	0.85	0.010

*STD* Standard deviation

^**^Indicates high significant *P* values ≤ 0.01

**Table 3:** Number of survived HeLa cells in 48 h post transfection

	**UT**	**Control**	**Anti-miR-141**	**miR-141 overexpression**
**Mean**	294000	232000	98000**	**319000**
**STD**	8485.28	67882.3	2828.43	**12727.9**
***P values***		0.328	0.01	**0.147**

*UT* Untreated cells, *STD* Standard deviation of three independent experiments

^**^Indicates high significant *P* values ≤ 0.01

### Microarray analysis represents miR-141-regulated genes, including KLRC and CAM gene family in HeLa cells.

**Fig. 2:**
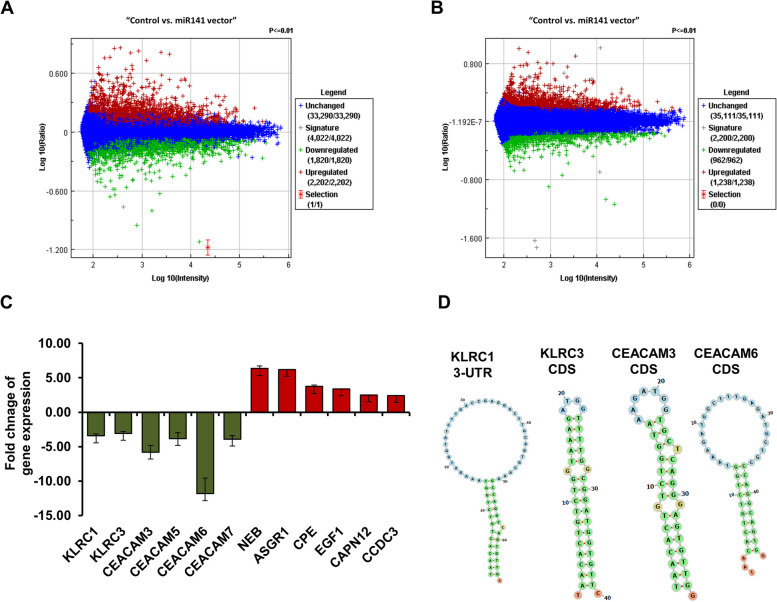
Microarray analysis of transfected HeLa cells with miR-141 overexpressing vector. **A** Microarray analysis of gene expression in HeLa cells transfected with miR-141 overexpression vs. cells transfected with the control vector using a miRNA library from Exiqon. **B** Microarray analysis of gene expression in HeLa cells transfected with miR-141 overexpression vs. cells transfected with the control vector using oligonucleotides from Ambion. The blue color indicates the sustained gene expression, the red indicates the upregulated genes, and the green indicates the downregulated genes. **C** The expression of the most relevant genes to cervical cancer indicates the upregulated genes in red columns and downregulated genes in green. Error bars indicate the STD between Exiqon and Ambion data. **D** The potential binding sites of miR-141 within the 3^−^ UTR of KLRC1 and coding sequences of KLRC3, CAM3, and that carried out in-silico by miRWalk online tool

As presented in Fig. 2A and B, the microarray data of transfected HeLa cells with pCMV-GFP-miR-141 construct showed that dozens of mRNA whose expression was negatively affected by the presence of high levels of miR-141 indicated by both the Exiqon and the Ambion chips. However, thousands of mRNA were not affected by the level of miR-141 in HeLa cells. On the contrary, the expression of many genes was elevated in the presence of these high levels of miR-141. Notably, mRNAs regulated by miR-141 were highly enriched for transcripts subject to cancer repression, and the miR-141-dependent regulation of many curtail tumor suppressor mRNAs, including the *KLRC1*, *KLRC3*, *CAM3*, *CAM5*, *CAM6*, *and CAM7* was approved (Fig. 2C). Alternatively, the most increased mRNAs were linked with the cancer progression and development, such as *NEB* encoding for nebulin protein, *ASGR1* encoding for asialoglycoprotein receptor 1, and *CPE* encoding for carboxypeptidase E (Fig. 2C). To check the direct interaction between miR-141 and identified genes by microarray analysis, the *in-silico miRWalk* tool was used to detect the possible binding site of miR-141 and targeted sequences. The docking interaction presented in Fig. 2D indicates the binding site of miR-141 within the 3^−^ untranslated region (3^–^UTR) of *KLRC1* and within the coding sequences (CDS) of *KLRC3*, *CAM3*, and *CAM6* targeted sequences. The seeding region, binding position, required energy, and docking score are presented in Table 4. These data indicate the integration of miR-141 in cervical cancer progression and suggest the possible regulatory role of miR-141 in *KLRC1*, *KLRC3*, *CAM3*, and *CAM6* gene expression in HeLa cells.

**Table 4:** The prediction information between miR-141 and targeted genes identified by microarray analysis using in-silico miRWalk tool

Ref. No	Gene symbol	Score	N pairings	Position	Binding site	Energy
**NM_001304448**	KLRC1	0.92	14	3^−^-URT	125–150	-18.5
**NM_007333**	KLRC3	0.84	16	CDS	675–706	-17
**NM_001277163**	CAM3	0.84	14	CDS	1575–1595	-18.4
**NM_002483**	CAM6	0.84	10	CDS	491–522	-16.5

### miR-141 modulates the expression of KLRC1 and KLRC3 in transfected HeLa cells

**Fig. 3:**
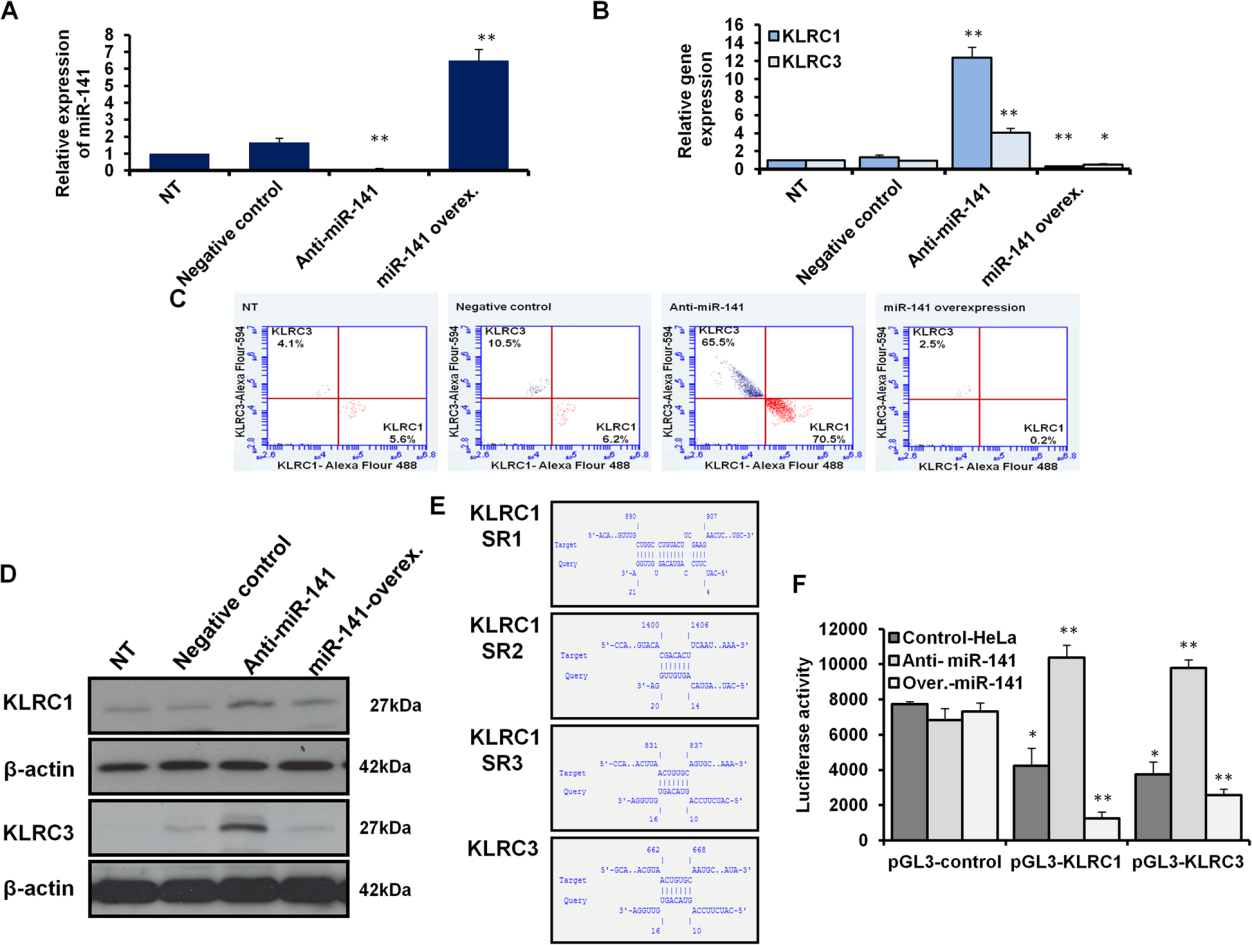
The relationship between miR-141 expression and KLRC gene expression profile of in HeLa cells. **A** Quantification of steady-state miR-141 in transfected HeLa cells compared with control-transfected and nontreated cells using qRT-PCR. **B** Relative gene expression of KLRC1 and KLRC3 in HeLa cells transfected with either specific inhibitor against miR-141 or miR-141 overexpression vector compared with control-transfected and nontreated cells using qRT-PCR. Error bars indicate the STD of three independent experiments. Student two-tailed *t*-test used for statistical analysis, (*) indicates *P-values* ≤ 0.05, and (**) indicates *P* ≤ 0.01. **C** Flow cytometric assay quantifies the kinetic proteins expression profile of KLRC1 (in red dots) and KLRC3 (in blue dots) in transfected cells compared with control-transfected and nontreated (NT) cells. **D** Western blot analysis reveals the protein expression level of KLRC1 and KLRC3 in transfected cells compared to control-transfected and nontreated cells. β-actin expression profile severed as an internal control. **E** Schematic representation of miR-141 binding site and seeding regions (SR) in KLRC1 and KLRC3 gene sequence indicated by IntaRNA program. **F** In HeLa cells pre-transfected with miR-141 overexpressing vector or specific inhibitor, the luciferase activities upon cotransfection with luciferase reporter constructs, pGL3-KLRC1 or pGL3-KLRC3 compared with cells cotransfected with pGL3-control vector. Error bars reveal the STD of three replicates. Student two-tailed *t*-test used for statistical analysis, (*) indicates *P-values* ≤ 0.05, and (**) indicates *P* ≤ 0.01

First, we confirmed the alteration of miR-141 expression level in transfected HeLa cells indicated by the fold change using qRT-PCR. The relative expression of miR-141 significantly upregulated sixfold change in cells transfected with the overexpression vector. In contrast, its expression level dramatically decreased in cells transfected with the miR-141 inhibitor compared to control-transfected cells (Fig. 3A and Table 4). To check the possible regulation of KLRC1 and KLRC3 expression by miR-141, the expression profile of KLRC1 and KLRC3 was quantified in transfected HeLa cells with the inhibitor antagonist miR-141 or miR-141 overexpression vector using qRT-PCR, flow cytometry, and immunoblotting assay. Consequently, the relative gene expression of both *KLRC1* and *KLRC3* strongly reduced in miR-141 transduced HeLa cells, while their expression increased in cells transfected with miR-141 inhibitor (Fig. 3B and Table 5). Furthermore, the kinetic protein expression of KLRC1 and KLRC3 markedly depleted in cells transduced miR-141, indicated by flow cytometry, since their expression has been detected in only 0.2% and 2.5% of stained cells (Fig. 3C). However, the protein expression profile of KLRC1 and KLRC3 showed an evident expression in more than 70% and 65% of stained cells transfected with the inhibitor antagonist miR-141 compared with control-transfected cells, as presented in Fig. 3C. The double-check of KLRC1 and KLRC3 protein expression profiles by western blot further confirmed the depletion of both proteins in HeLa cells transfected with the miR-141 overexpressing vector compared with control-transfected and nontreated cells. In contrast, they showed full expression in HeLa cells transfected with the inhibitor antagonist miR-141 (Fig. 3D and Supp. Figures 1, and 2). To identify the binding site of miR-141 on the *KLRC* genes sequence, the IntaRNA program was used. Interestingly, three binding sites have been detected in the coding sequence of *KLRC1*, which interfere with the mature miR-141 by a required energy -8.39, -5.75, and -0.21, respectively (Fig. 3E and Table 6). KLRC3 showed one binding site within its coding sequence with a required energy -5.42 (Fig. 3E and Table 6). Furthermore, we cloned the seeding region of potentially targeted sequences in the pGL3 luciferase reporter vector with SV40 promoter to validate the direct interaction between miR-141 and *KLRC1* and *KLRC3* coding sequences (Supp. Figure 3). Interestingly, the luciferase activity significantly decreased in HeLa cells co-transfected with the miR-141 overexpression vector and the luciferase reporter construct (pGL3-KLRC1 and pGL3-KLRC1), including the binding site sequences of *KLRC1* and *KLRC3* cloned upstream of luciferase reporter gene. Meanwhile, the luciferase activities markedly increased in cells co-transfected with the specific inhibitor antagonist miR-141 and the luciferase reporter constructs with seeding regions of targeted genes (Fig. 3F). These findings indicate the first evidence concerning regulating *KLRC1* and *KLRC3* gene expression by miR-141 in cervical cancer cells.

**Table 5:** Quantification analysis of miR-141, KLRC1, and KLRC3 in transfected HeLa cells

**Genes**	**Condition**	**Expression fold changes**	**STD**	***P*-values**
**miR-141**	NT	1.00	0.00	
	Control	1.66	0.25	0.065
	Anti-miR-141	0.12**	0.01	0.0001
	miR-141 over	6.49**	0.67	0.007
**KLRC1**	NT	1.00	0.00	
	Control	1.15	0.17	0.35
	Anti-miR-141	6.51*	1.37	0.025
	miR-141 over	0.20**	0.12	0.010
**KLRC3**	NT	1	0.00	
	Control	1.26	0.44	0.49
	Anti-miR-141	4.82**	0.59	0.010
	miR-141 over	0.28**	0.01	0.002
**CAM3**	NT	1	0.00	
	Control	1.15	0.17	0.35
	Anti-miR-141	6.51**	1.37	0.010
	miR-141 over	0.20**	0.12	0.002
**CAM6**	NT	1	0.00	
	Control	1.25	0.40	0.45
	Anti-miR-141	4.85**	0.50	0.010
	miR-141 over	0.25**	0.03	0.003

*NT* nontreated cells, *STD* Standard deviation of three independent experiments

^*^Indicates significant *P* values ≤ 0.05

^**^Indicates high significant *P* values ≤ 0.01

**Table 6:** detailed of selected interaction between miR-141 and targeted genes

**Target (T)**	**Start (T)**	**End(T)**	**Query (Q)**	**Start (Q)**	**End(Q)**	**Energy**
**KLRC1**	890	907	Hsa-miR-141	4	21	**-8.39**
**KLRC1**	1077	1083	Hsa-miR-141	10	16	**-5.75**
**KLRC1**	1126	1132	Hsa-miR-141	16	22	**-0.21**
**KLRC3**	662	668	Hsa-miR-141	10	16	**-5.42**
**CAM3**	494	503	Hsa-miR-141	5	14	**-5.39**
**CAM6**	512	521	Hsa-miR-141	5	14	**-7.85**
**CAM6**	1656	1669	Hsa-miR-141	6	21	**-4.21**

### miR-141 significantly inhibits the expression profile of CAM3 and CAM6 in transfected HeLa cells

**Fig. 4:**
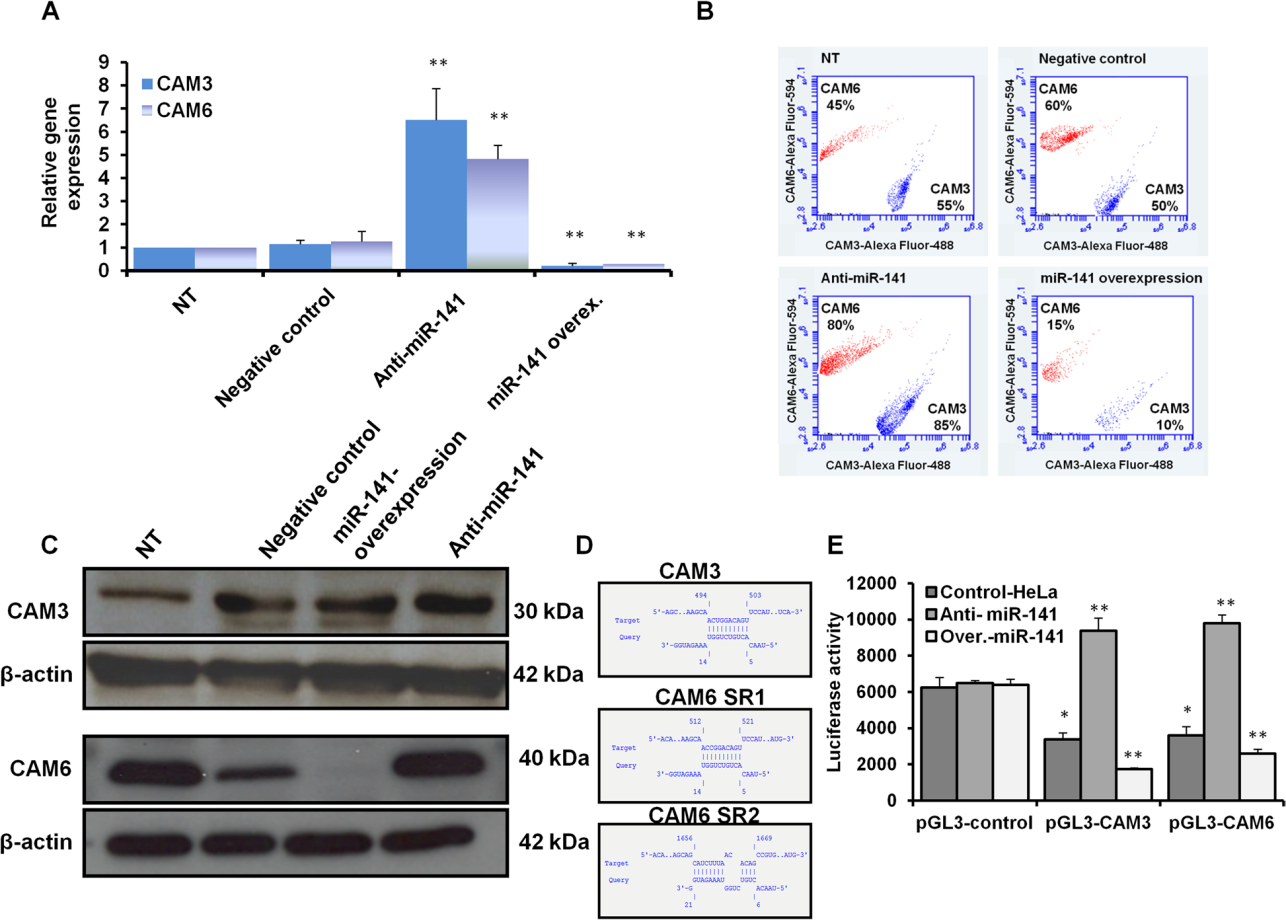
The correlation between the level of miR-141 and CAM gene expression in HeLa cells. **A** Relative gene expression of CAM3 and CAM6 in HeLa cells transfected with the inhibitor against miR-141 or miR-141 overexpression vector compared with control-transfected and nontreated cells using qRT-PCR. Error bars indicate the STD of three independent experiments. Student two-tailed *t*-test used for statistical analysis**,** (*) indicates *P-values* ≤ 0.05, and (**) indicates *P* ≤ 0.01. **B** Flow cytometric assay quantifies the kinetic proteins expression profile of CAM3 (the blue dots) and CAM6 (the red dots) in transfected cells compared with control-transfected and nontreated (NT) cells. **C** Western blot analysis shows the protein expression of CAM3 and CAM6 in transfected cells compared to control-transfected and nontreated cells. β-actin expression severed as an internal control. **D** Schematic representation of miR-141- binding site and seeding regions (SR) in CAM3 and CAM6 gene sequence indicated by IntaRNA program. **E** In HeLa cells pre-transfected with miR-141 overexpressing vector or specific inhibitor, the luciferase activities upon cotransfection with luciferase reporter constructs, pGL3-CAM3 or pGL3-CAM6 compared with cells cotransfected with pGL3-control vector. Error bars reveal the STD of three replicates. Student two-tailed *t*-test used for statistical analysis, (*) indicates *P-values* ≤ 0.05, and (**) indicates *P* ≤ 0.01

To investigate the connection between miR-141 expression level and *CAMs* gene expression, the expression profile of CAM3 and CAM6 has been achieved in transfected HeLa cells with either miR-141 inhibitor or the overexpression vector. Interestingly, the relative gene expression of *CAM3* and *CAM6* strongly reduced in HeLa cells transduced the miR-141, while their expression resorted in cells transfected with the miR-141 inhibitor (Fig. 4A and Table 5). Furthermore, the kinetic protein expression of CAM3 and CAM6 markedly depleted in cells transduced the miR-141 since their expression has been detected in only 10% and 15% of stained cells (Fig. 4B). However, the protein expression profile of both CAM3 and CAM6 showed a noticeable recovery in more than 85% and 80% of stained cells transfected with the miR-141 inhibitor compared with control-transfected cells, as presented in Fig. 4B. The double-check of CAM3 and CAM6 protein expression by immunoblotting confirmed the depletion of CAM3 and CAM6 proteins in HeLa cells transfected with the miR-141 overexpressing vector compared with control-transfected and nontreated cells (Fig. 4C). In contrast, their expression showed marked recovery in HeLa cells transfected with the inhibitor antagonist miR-141 (Fig. 4C and Supp. Figures 4 and 5). To identify the seeding region on the CAM genes sequence and miR-141, the IntaRNA program was used to recognize the binding sites of *CAM3*, *CAM6*, and the mature miR-141. Interestingly, the same binding site in the coding sequence of *CAM3* and *CAM6* interferes with the mature miR-141 with the required energy of -5.39 and -7.85, respectively (Fig. 4D and Table 6). However, *CAM6* showed another binding site by the end of its coding sequence that interferes with 15 nucleotides in the mature miR-141 with the required energy of -4.25 (Fig. 4D and Table 6). As shown in Supp. Figure 6, the cloned luciferase constructs, including the seeding regions of *CAM3* and *CAM6*, were used to transfect HeLa cells that were pre-transfected with either the miR-141 overexpressing vector or its specific inhibitor. The luciferase activity was significantly reduced in cells pre-transfected with miR-141 overexpression vector while increased dramatically in cells pre-transfected with the specific inhibitor antagonist miR-141 compared with control transfected cells (Fig. 4E). These findings from prediction tools and cloning vectors indicate the direct interaction of the mature miR-141 and the *CAM* genes coding sequence. Together, these data suggest the first evidence concerning the negative correlation between miR-141 and *CAM* gene expression.

### Deception of miR-141 successfully modifies the production of TFG-β and IL-8 in transfected HeLa cells

**Fig. 5:**
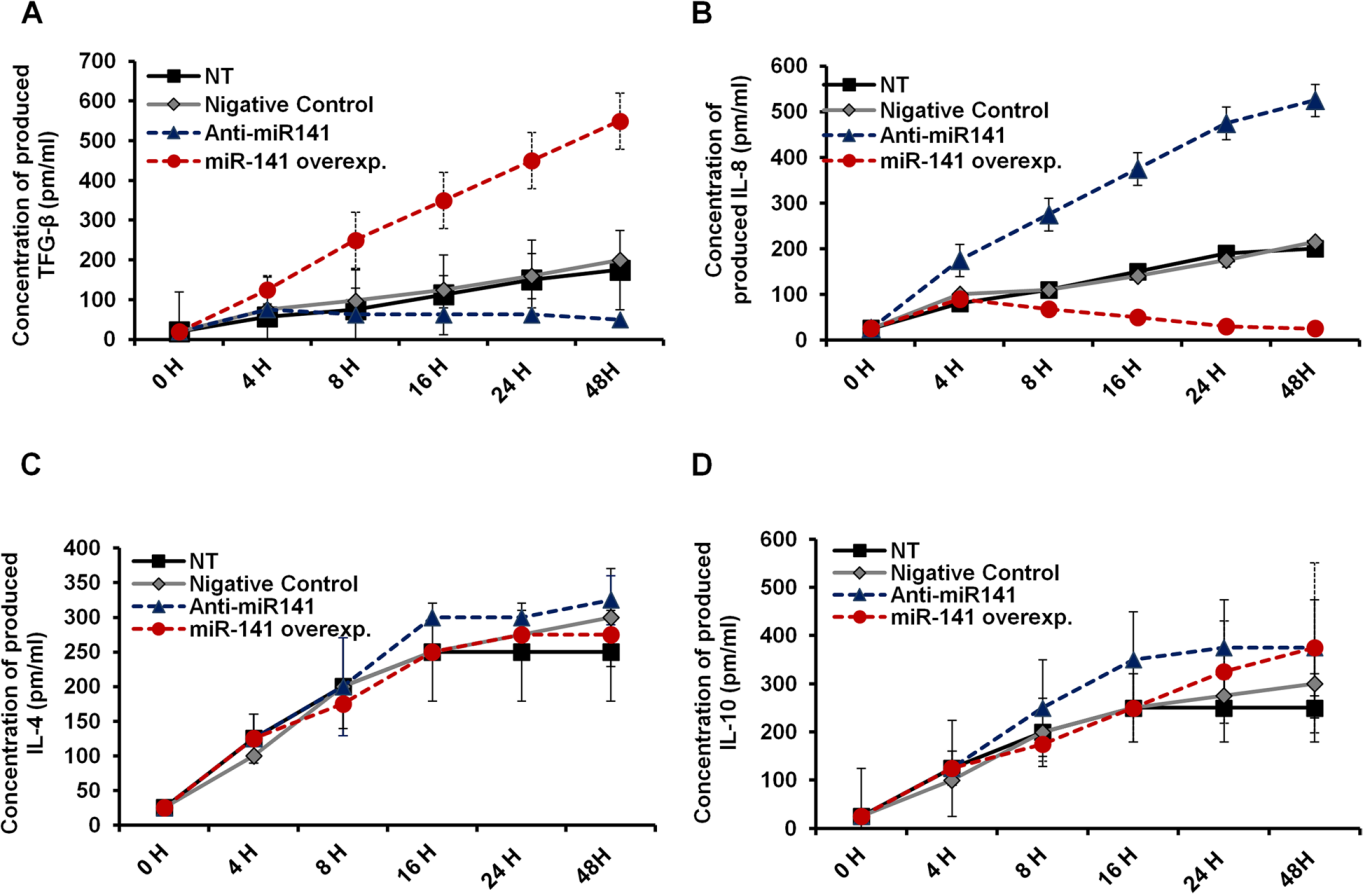
Levels of produced inflammatory cytokines in transfected HeLa cells. **A** The TGF-β concentration (pm/ml) produced in the fluid media of transfected HeLa cells in response to the expression level of miR-141 at the indicated time points. **B** IL-8 concentration (pm/ml) in the culture media of transfected HeLa cells simultaneously post-transfection. **C** and **D** At the same time points, the concentration of IL-4 and IL-10 in the fluid media of transfected HeLa cells points to post-transfection, respectively

To investigate the relationship between miR-141 expression level and cytokine production, the concentration of secreted TFG-β, IL-8, IL-4, and IL-10 was monitored in transfected HeLa cells in a time-course experiment. As shown in Fig. 5A, the amount of produced TFG-β increased in cells transfected with the miR-141 overexpression vector in a time-dependent manner, reached 500 pm/ml at 48 h post-transfection. The level of TFG-β markedly decreased in cells transfected with the miR-141 inhibitor compared with control transfected cells. In contrast, the level of produced IL-8 significantly decreased in the miR-141 transduced cells while markedly increased up to 500 pm/ml in cells transfected with the inhibitor antagonist miR-141 (Fig. 5B). Meanwhile, the concentration of IL-4 and IL-10 showed neglected differences in transfected HeLa cells compared with non-transfected (NT) and control-transfected cells (Fig. 5C and D). These data indicate that inhibition of miR-141 expression can adjust the production level of TFG-β and increase the produced IL-8 in HeLa cells without affecting the anti-inflammatory cytokines IL-4 and IL-10.

## Discussion

As a member of the miR-200 family, miR-141 is typically expressed in various malignant tumors, contributing to many cellular processes such as cell proliferation, epithelial-mesenchymal transition (EMT), invasion, metastasis, and drug resistance [19]. As indicated in a pilot study, miR-141 was significantly expressed in cancerous patients, particularly patients with metastatic stage, compared to non-metastatic patients [36]. MiR-141 has been identified as one of the top ten upregulated miRNAs accompanied by overexpression of *Drosha*, the miRNA processor, in clinical samples obtained from patients with cervical cancer [37]. Notably, a recent study indicated the overexpression of miR-141 and miR-340 in cervical squamous cell carcinoma (CSCC), suggesting a potential role of these miRNAs in regulating the tumor suppressor PTEN gene [20]. A recent study highlighted the interactions between circulating RNAs (circRNA) and miR-141 with emphasis on the biological role of circRNA, miR-141, and mRNA networks as a novel target for anti-cancer therapies, including cervical cancer therapy [38]. Recent evidence indicated that miR-141 is upregulated in cervical cancer and was negatively associated with the prognosis of patients with cervical cancer. Antagonist miR-141 expression alleviated apoptotic signaling and inhibited the cell proliferation, migration, and invasion of C33A and HeLa cells [39]. In cervical pre-cancerous tissue, the expressions of miRNAs, including miR-141, miR145, and miR-375, were dysregulated and responsible for DNA damage response and cell growth response induced by human papillomavirus (HPV) during the early transformation of the cervical cells [40]. Overall, miR-141 is aberrantly expressed in many human malignant tumors, participating in various cellular processes, including epithelial-mesenchymal transition (EMT), proliferation, migration, invasion, and drug resistance, indicating the possible future of miR-141 as a potential diagnostic and prognostic parameter as well as a therapeutic target in clinical applications [19]. The cellular targeted genes by miR-141 are mostly related to tumor suppressor mechanism; however, none of these genes provide a plausible explanation for the impact of this miRNA on cancer metastasis. Yet, miR-378 was found to play a potential role in the metastatic stage via targeting the autophagy related-12 (Atg12) in cervical cancer cells [41]. Here, we exhibit the overexpression of miR-141 in cervical cancer cell lines, HeLa cells, and C-33A cells, compared to the normal cervical cells, HCK1T cell line. Similarly, recent evidence indicated the upregulation of miR-141 in patients with gallbladder cancer (GBC) and reported the value of miR-141 expression level as an early diagnostic and prognostic biomarker in gallbladder cancer [42]. Furthermore, the microarray data provided here clearly shows the ability of miR-141 to regulate a variety of gene expressions in transfected HeLa cells. Among the targeted mRNAs affected almost three-fold in miR-141 transduced cells were killer cell lectin-like receptor C1 (*KLRC1*) and *KLRC3* mRNAs. In addition, the *CAM* gene family, including *CAM3*, *CAM5, CAM6*, and *CAM7*, dramatically down-regulated in miR-141 transduced cells via direct interaction with miR-141 in their coding sequence. In contrast, some identified mRNAs significantly upregulated in miR-141 transduced cells, such as *NEB* encoding for nebulin protein, *ASGR1* encoding for asialoglycoprotein receptor 1, and *CPE* encoding for carboxypeptidase E. Most of them play a potential role in tumorigenesis and cancer diagnosis, particularly in hepatic patients [43, 44].

Notably, the anti-tumor activities of natural killer (NK) cell family receptors, including *KLRC1* and *KLRC3,* have been reported in different cancers and cancer phases, particularly in metastasis [45]. For instance, the *KLRC2* encoding for immunoreceptor NKp44 and expressed in NK and lymphoid cells was shown to trigger the secretion of tumor necrosis factor-alpha (TNF-α) and interferon-gamma (IFN-γ) that stimulate cell growth arrest [46]. Thus, the microarray data suggested that the upregulation of miR-141 modulates the expression of the *KLRC* gene family to facilitate cancer cell proliferation and tumor resistance to immune cells. On the other hand, cell adhesion proteins (CEA) belong to the immunoglobulin family that are expressed in a wide range of tissues and cell types. CEA molecules exert context-dependent activating or inhibitory effects on cancer cell growth [47]. Among CEA molecules, the CAMs protein family are transmembrane molecules with an extracellular and cytoplasmic domain. The cytoplasmic domain of CAM contains immunoreceptor tyrosine-based inhibitory motifs. It acts as a co-receptor that regulates the activation of different types of receptors such as vascular endothelial growth factor receptor 2 (VEGFR2), T, and B cell receptors [48]. Most importantly, the contribution of the *CAM* gene family in the early phases of cancer and solid tumor as tumor suppressor genes has been reported. Its depletion is lately linked to the malignancy transformation, angiogenesis, and metastasis of cancerous cells [49].

The TFG-β production signaling has been connected with the *CAM* gene expression family in colon cancer, therapy possibly regulating cell adhesion and metastasis in cancer [50]. Evidence indicated that *CAM5* and *CAM6's* expression patterns correlates with the TFG-β signaling pathway [51]. Based on this, we hypothesized that upregulation of miR-141 in cervical cancer cells is required to create the atmosphere for metastatic progress via targeting the *CAM* gene family and independently stimulating the production of TFG-β. Alternatively, targeting miR-141 in HeLa cells with a specific inhibitor restored *CAM* gene expression, reduced TFG-β production, and stimulated IL-8 secretion contributing to programmed cell death (PCD). As evidence supporting our hypothesis, a very recent study reported the role of miR-498 in supporting cell proliferation, migration, and epithelial to mesenchymal alteration in gastric cancer via targeting CAM5 [52]. Another study indicated the association between CAM's mutation and aberrant expression and inherited colorectal cancer and breast cancer risk [53]. Interestingly, the undetectable expression of CAM1 was observed in cervical carcinoma; however, its expression increased in women with high-grade squamous intraepithelial lesions (SIL) [54]. Therefore, our findings declare that the depletion of the *KLRC* and *CAM* gene expression in cervical cancer is due to the upregulation of miR-141 to facilitate cancer cell proliferation and metastasis.

## Conclusion

The present study shows the upregulation of miR-141 in cervical cancer HeLa and C-33A cells compared to the normal cervical HCK1T cells. Microarray analysis of transfected HeLa cells with miR-141 overexpression vector reveals that miR-141 is implicated in cancer cell proliferation and metastasis via targeting *KLRC* and *CAM *gene family expression profiles, respectively. Moreover, miR-141 transduced HeLa cells further confirm the regulation of *KLRC1*, *KLRC3*, *CAM3*, and *CAM6* gene expression by miR-141 at both RNA and protein levels. This regulation was accompanied by an increasing level of produced transforming growth factor alpha (TFG-α) and decreasing levels of IL-8 production. In contrast, transfected HeLa cells with an inhibitor antagonist miR-141 expression provide marked restoration of *KLRC* and *CAM* gene expression and increase the production of IL-8. Investigation of the seeding region in *KLRC* and *CAM* gene sequences suggested binding sites interfered with miR-141 in their coding sequences. These data indicate that miR-141 is involved in cervical cancer progression and metastasis via targeting related factors such as *KLRC* and *CAM* gene expression.

### Supplementary Information

Supplementary Material 1

## Data Availability

The data supporting these findings are included in the main manuscript and supplementary file. The whole microarray results are available from the corresponding author upon reasonable request.

## References

[1] Ferlay J, Colombet M, Soerjomataram I, Mathers C, Parkin DM, Piñeros M, et al. Estimating the global cancer incidence and mortality in 2018: GLOBOCAN sources and methods. 2018. 10.1002/ijc.31937. 10.1002/ijc.31937 30350310

[2] Arbyn M, Weiderpass E, Bruni L, de Sanjosé S, Saraiya M, Ferlay J, et al. Estimates of incidence and mortality of cervical cancer in 2018: a worldwide analysis. Lancet Glob Heal. 2020;8:e191-203. 10.1016/S2214-109X(19)30482-6 PMC7025157 31812369

[3] Hull R, Mbele M, Makhafola T, Hicks C, Wang SM, Reis RM, et al. Cervical cancer in low and middle-income countries (Review). Oncol Lett. 2020;20:2058–74. 32782524 10.3892/ol.2020.11754 PMC7400218

[4] Balcik-Ercin P, Cetin M, Yalim-Camci I, Uygur T, Yagci T. Hepatocellular Carcinoma Cells with Downregulated ZEB2 Become Resistant to Resveratrol by Concomitant Induction of ABCG2 Expression. Mol Biol. 2020;54:75–81. 10.1134/S0026893320010033 32163392

[5] Kamiya T, Seow SV, Wong D, Robinson M, Campana D. Blocking expression of inhibitory receptor NKG2A overcomes tumor resistance to NK cells. J Clin Invest. 2019;129:2094–106. 30860984 10.1172/JCI123955 PMC6486333

[6] Hoeben A, Landuyt B, Highley MS, Wildiers H, Van Oosterom AT, De Bruijn EA. Vascular Endothelial Growth Factor and Angiogenesis. Pharmacol Rev. 2004;56:549–80. 15602010 10.1124/pr.56.4.3

[7] Liu Q, Zhang H, Jiang X, Qian C, Liu Z, Luo D. Factors involved in cancer metastasis: a better understanding to “seed and soil” hypothesis. Mol Cancer. 2017;16:176. 29197379 10.1186/s12943-017-0742-4 PMC5712107

[8] Maher E, Gedawy G, Fathy W, Farouk S, El Maksoud AA, Guirgis AA, et al. Hsa-miR-21-mediated cell death and tumor metastases: A potential dual response during colorectal cancer development. Middle East J Cancer. 2020;4:483–92.

[9] Ling H, Fabbri M, Calin GA. MicroRNAs and other non-coding RNAs as targets for anticancer drug development. Nat Rev Drug Discov. 2013;12:847–65. 24172333 10.1038/nrd4140 PMC4548803

[10] MacFarlane L-A, R. Murphy P. MicroRNA: Biogenesis, function and role in cancer. Curr Genomics. 2010. 10.2174/138920210793175895. 10.2174/138920210793175895 PMC3048316 21532838

[11] Tazi MF, Dakhlallah DA, Caution K, Gerber MM, Chang SW, Khalil H, et al. Elevated Mirc1/Mir17-92 cluster expression negatively regulates autophagy and CFTR (cystic fibrosis transmembrane conductance regulator) function in CF macrophages. Autophagy. 2016. 10.1080/15548627.2016.1217370. 27541364 10.1080/15548627.2016.1217370 PMC5103351

[12] Schulman BRM, Esquela-Kerscher A, Slack FJ. Reciprocal expression of lin-41 and the microRNAs let-7 and mir-125 during mouse embryogenesis. Dev Dyn. 2005;234:1046–54. 16247770 10.1002/dvdy.20599 PMC2596717

[13] Ying S-Y, Chang DC, Lin S-L. The MicroRNA (miRNA): Overview of the RNA Genes that Modulate Gene Function. Mol Biotechnol. 2008;38:257–68. 17999201 10.1007/s12033-007-9013-8 PMC7091389

[14] Abbas M, Mehdi A, Khan FH, Verma S, Ahmad A, Khatoon F, et al. Role of miRNAs in cervical cancer: A comprehensive novel approach from pathogenesis to therapy. J Gynecol Obstet Hum Reprod. 2021;50:102159. 33965650 10.1016/j.jogoh.2021.102159

[15] Banno K, Iida M, Yanokura M, Kisu I, Iwata T, Tominaga E, et al. MicroRNA in cervical cancer: OncomiRs and tumor suppressor miRs in diagnosis and treatment. Sci World J. 2014;2014(2):178075. 10.1155/2014/178075 PMC3910129 24516357

[16] Yao Q, Xu H, Zhang Q-Q, Zhou H, Qu L-H. MicroRNA-21 promotes cell proliferation and down-regulates the expression of programmed cell death 4 (PDCD4) in HeLa cervical carcinoma cells. Biochem Biophys Res Commun. 2009;388:539–42. 19682430 10.1016/j.bbrc.2009.08.044

[17] Yao T, Lin Z. MiR-21 is involved in cervical squamous cell tumorigenesis and regulates CCL20. Biochim Biophys Acta - Mol Basis Dis. 2012;1822:248–60. 10.1016/j.bbadis.2011.09.018 22001440

[18] Xu X-M, Wang X-B, Chen M-M, Liu T, Li Y-X, Jia W-H, et al. RETRACTED: MicroRNA-19a and -19b regulate cervical carcinoma cell proliferation and invasion by targeting CUL5. Cancer Lett. 2012;322:148–58. 22561557 10.1016/j.canlet.2012.02.038

[19] Gao Y, Feng B, Han S, Zhang K, Chen J, Li C, et al. The Roles of MicroRNA-141 in Human Cancers: From Diagnosis to Treatment. Cell Physiol Biochem. 2016;38:427–48. 26828359 10.1159/000438641

[20] Li W, Yang B, Li Y, Wang C, Fang X. Significance of miR-141 and miR-340 in cervical squamous cell carcinoma. Open Med. 2021;16:864–72. 10.1515/med-2021-0281 PMC8209411 34179503

[21] El-Fadl HMA, Hagag NM, El-Shafei RA, Khayri MH, El-Gedawy G, El MAIA, et al. Effective Targeting of Raf-1 and Its Associated Autophagy by Novel Extracted Peptide for Treating Breast Cancer Cells. Front Oncol. 2021;11:3317. 10.3389/fonc.2021.682596 PMC8430328 34513674

[22] Guirgis SA, El-Halfawy KA, Alalem M, Khalil H. Legionellapneumophila induces methylomic changes in ten-eleven translocation to ensure bacterial reproduction in human lung epithelial cells. J Med Microbiol. 2023;72(3):001676. 10.1099/jmm.0.001676 36927577

[23] Alalem M, Dabous E, Awad AM, Alalem N, Guirgis AA, El-Masry S, et al. Influenza a virus regulates interferon signaling and its associated genes; MxA and STAT3 by cellular miR-141 to ensure viral replication. Virol J. 2023;20:183. 37596622 10.1186/s12985-023-02146-4 PMC10439583

[24] Abd El Maksoud AI, Elebeedy D, Abass NH, Awad AM, Nasr GM, Roshdy T, et al. Methylomic changes of autophagy-related genes by Legionella Effector Lpg2936 in infected macrophages. Front Cell Dev Biol. 2020;7:390. 10.3389/fcell.2019.00390 PMC6999459 32064256

[25] Hamouda RA, Abd El Maksoud AI, Wageed M, Alotaibi AS, Elebeedy D, Khalil H, et al. Characterization and anticancer activity of biosynthesized Au/Cellulose nanocomposite from Chlorella vulgaris. Polymers (Basel). 2022;23(7):2531. 10.3390/polym13193340 PMC8512388 34641156

[26] Mohamed E-SA, Bassiouny K, Alshambky AA, Khalil H. Anticancer Properties of N,N-dibenzylasparagine as an Asparagine (Asp) analog, Using colon cancer Caco-2 Cell Line. Asian Pacific J Cancer Prev. 2022;23(2531):40. 10.31557/APJCP.2022.23.7.2531 PMC9727330 35901362

[27] Awad AM, Dabous E, Alalem M, Alalem N, Nasr ME, Elawdan KA, et al. MicroRNA-141-regulated KLK10 and TNFSF-15 gene expression in hepatoblastoma cells as a novel mechanism in liver carcinogenesis. Sci Rep. 2024;14:13492. 38866875 10.1038/s41598-024-63223-4 PMC11169620

[28] Iglesias-Ussel M, Marchionni L, Romerio F. Isolation of microarray-quality RNA from primary human cells after intracellular immunostaining and fluorescence-activated cell sorting. J Immunol Methods. 2013;391:22–30. 23434645 10.1016/j.jim.2013.02.003 PMC3627819

[29] Elawdan KA, Farouk S, Aref S, Shoaib H, El-Razik MA, Abbas NH, et al. Association of vitamin B12/ferritin deficiency in cancer patients with methylomic changes at promotors of TET methylcytosine dioxygenases. Biomark Med. 2022;16:959–70. 36052661 10.2217/bmm-2022-0158

[30] Abd El Maksoud AI, Taher RF, Gaara AH, Abdelrazik E, Keshk OS, Elawdan KA, et al. Selective regulation of B-Raf dependent K-Ras/mitogen-activated protein by natural occurring multi-kinase inhibitors in cancer cells. Front Oncol. 2019;9:1220. 10.3389/fonc.2019.01220 PMC6861212 31781509

[31] Taher RF, Al-Karmalawy AA, Abd El Maksoud AI, Hassan A, El-Khrisy E-DA, et al. Two new flavonoids and anticancer activity of Hymenosporum flavum: in vitro and molecular docking studies. J Herbmed Pharmacol. 2021;10:443–58. 10.34172/jhp.2021.52

[32] Fekry T, Salem MF, Abd-Elaziz AA, Muawia S, Naguib YM, Khalil H. Anticancer Properties of Selenium-Enriched Oyster Culinary-Medicinal Mushroom, Pleurotus ostreatus (Agaricomycetes), in Colon Cancer In Vitro. Int J Med Mushrooms. 2022;24:1–20. 36374945 10.1615/IntJMedMushrooms.2022045181

[33] Elimam H, El-Say KM, Cybulsky AV, Khalil H. Regulation of Autophagy Progress via Lysosomal Depletion by Fluvastatin Nanoparticle Treatment in Breast Cancer Cells. ACS Omega. 2020. 10.1021/acsomega.0c01618. 32637822 10.1021/acsomega.0c01618 PMC7331036

[34] Rao X, Huang X, Zhou Z, Lin X. An improvement of the2–delta delta CT) method for quantitative real-time polymerase chain reaction data analysis. Biostat Bioinforma Biomath. 2013. 10.1016/j.micinf.2011.07.011.Innate. 25558171 10.1016/j.micinf.2011.07.011.Innate PMC4280562

[35] Khalil H, Arfa M, El-Masrey S, EL-Sherbini S, Abd-Elaziz A. Single nucleotide polymorphisms of interleukins associated with hepatitis C virus infection in Egypt. J Infect Dev Ctries. 2017;11:261–8. 28368861 10.3855/jidc.8127

[36] Ali R, El Tabbakh S, El Delgawy W, Kotb A, Desouky MN. microRNA-141 as a diagnostic and prognostic biomarker for prostate cancer in Egyptian population: Pilot study. African J Urol. 2018;24:347–52. 10.1016/j.afju.2018.11.006

[37] Muralidhar B, Winder D, Murray M, Palmer R, Barbosa-Morais N, Saini H, et al. Functional evidence that Drosha overexpression in cervical squamous cell carcinoma affects cell phenotype and microRNA profiles. J Pathol. 2011;224:496–507. 21590768 10.1002/path.2898

[38] Guz M, Jeleniewicz W, Cybulski M. Interactions between circRNAs and miR-141 in Cancer: from pathogenesis to diagnosis and therapy. Int J Mol Sci. 2023;24(14):11861. 10.3390/ijms241411861 PMC10380543 37511619

[39] Ni Z, Shen Y, Wang W, Cheng X, Fu Y. miR-141-5p Affects the Cell Proliferation and Apoptosis by Targeting BTG1 in Cervical Cancer. Cancer Biother Radiopharm. 2021. 10.1089/cbr.2021.0227. 34767738 10.1089/cbr.2021.0227

[40] Choi P-W, Liu TL, Wong CW, Liu SK, Lum Y-L, Ming W-K. The dysregulation of MicroRNAs in the development of cervical pre-cancer—an update. Int J Molecular Sci. 2022;23(13):7126. 10.3390/ijms23137126 PMC9266862 35806128

[41] Tan D, Zhou C, Han S, Hou X, Kang S, Zhang Y. MicroRNA-378 enhances migration and invasion in cervical cancer by directly targeting autophagy-related protein 12. Mol Med Rep. 2018;17:6319–26. 29488616 10.3892/mmr.2018.8645 PMC5928611

[42] Yang G, Lu Z, Meng F, Wan Y, Zhang L, Xu Q, et al. Circulating miR-141 as a potential biomarker for diagnosis, prognosis and therapeutic targets in gallbladder cancer. Sci Rep. 2022;12:10072. 35710767 10.1038/s41598-022-13430-8 PMC9203542

[43] Ji L, Wu H-T, Qin X-Y, Lan R. Dissecting carboxypeptidase E: properties, functions and pathophysiological roles in disease. Endocr Connect. 2017;6:R18-38. 28348001 10.1530/EC-17-0020 PMC5434747

[44] Harris RL, van den Berg CW, Bowen DJ. *ASGR1* and *ASGR2*, the Genes that Encode the Asialoglycoprotein Receptor (Ashwell Receptor), are expressed in peripheral blood monocytes and show interindividual differences in transcript profile. Mol Biol Int. 2012;2012:283974. 22919488 10.1155/2012/283974 PMC3419429

[45] Sun Y, Sedgwick AJ, Palarasah Y, Mangiola S, Barrow AD. A Transcriptional signature of PDGF-DD activated natural killer cells predicts more favorable prognosis in low-grade glioma. Front Immunol. 2021;12:668391. 10.3389/fimmu.2021.668391 PMC8444979 34539622

[46] Barrow AD, Edeling MA, Trifonov V, Luo J, Goyal P, Bohl B, et al. Natural Killer Cells Control Tumor Growth by Sensing a Growth Factor. Cell. 2018;172:534-548.e19. 29275861 10.1016/j.cell.2017.11.037 PMC6684025

[47] Rueckschloss U, Kuerten S, Ergün S. The role of CEA-related cell adhesion molecule-1 (CEACAM1) in vascular homeostasis. Histochem Cell Biol. 2016;146:657–71. 27695943 10.1007/s00418-016-1505-9

[48] Kilic N, Oliveira-Ferrer L, Neshat-Vahid S, Irmak S, Obst-Pernberg K, Wurmbach J-H, et al. Lymphatic reprogramming of microvascular endothelial cells by CEA-related cell adhesion molecule-1 via interaction with VEGFR-3 and Prox1. Blood. 2007;110:4223–33. 17761831 10.1182/blood-2007-06-097592

[49] Calinescu A, Turcu G, Nedelcu RI, Brinzea A, Hodorogea A, Antohe M, et al. On the Dual Role of Carcinoembryonic Antigen-Related Cell Adhesion Molecule 1 (CEACAM1) in Human Malignancies. J Immunol Res. 2018;2018:7169081. 30406153 10.1155/2018/7169081 PMC6204181

[50] Li Y, Cao H, Jiao Z, Pakala SB, Sirigiri DNR, Li W, et al. Carcinoembryonic Antigen Interacts with TGF-β Receptor and Inhibits TGF-β Signaling in Colorectal Cancers. Cancer Res. 2010;70:8159–68. 20889724 10.1158/0008-5472.CAN-10-1073 PMC3001246

[51] Han S-U, Kwak T-H, Her KH, Cho Y-H, Choi C, Lee H-J, et al. CEACAM5 and CEACAM6 are major target genes for Smad3-mediated TGF-β signaling. Oncogene. 2008;27:675–83. 17653079 10.1038/sj.onc.1210686

[52] Zhang L, Zhang C, Liu N. CEACAM5 targeted by miR-498 promotes cell proliferation, migration and epithelial to mesenchymal transition in gastric cancer. Transl Oncol. 2022;24:101491. 35882167 10.1016/j.tranon.2022.101491 PMC9309501

[53] Huskey ALW, Merner ND. An investigation into the role of inherited CEACAM gene family variants and colorectal cancer risk. BMC Res Notes. 2022;15:26. 35115044 10.1186/s13104-022-05907-6 PMC8815132

[54] Albarran-Somoza B, Franco-Topete R, Delgado-Rizo V, Cerda-Camacho F, Acosta-Jimenez L, Lopez-Botet M, et al. CEACAM1 in Cervical Cancer and Precursor Lesions: Association With Human Papillomavirus Infection. J Histochem Cytochem. 2006;54:1393–9. 16924126 10.1369/jhc.6A6921.2006 PMC3958116

