# Supplementary material for: Regulation of KLRC and Ceacam gene expression by miR-141 supports cell proliferation and metastasis in cervical cancer cells
Source: BMC Cancer. 2024 Sep 3;24:1091. doi: 10.1186/s12885-024-12794-6 (PMC11370040; doi:10.1186/s12885-024-12794-6)
Supplement: Supplementary file 1 — Supplementary Material 1 [file 12885_2024_12794_MOESM1_ESM.docx]

**Supplementary data**

**
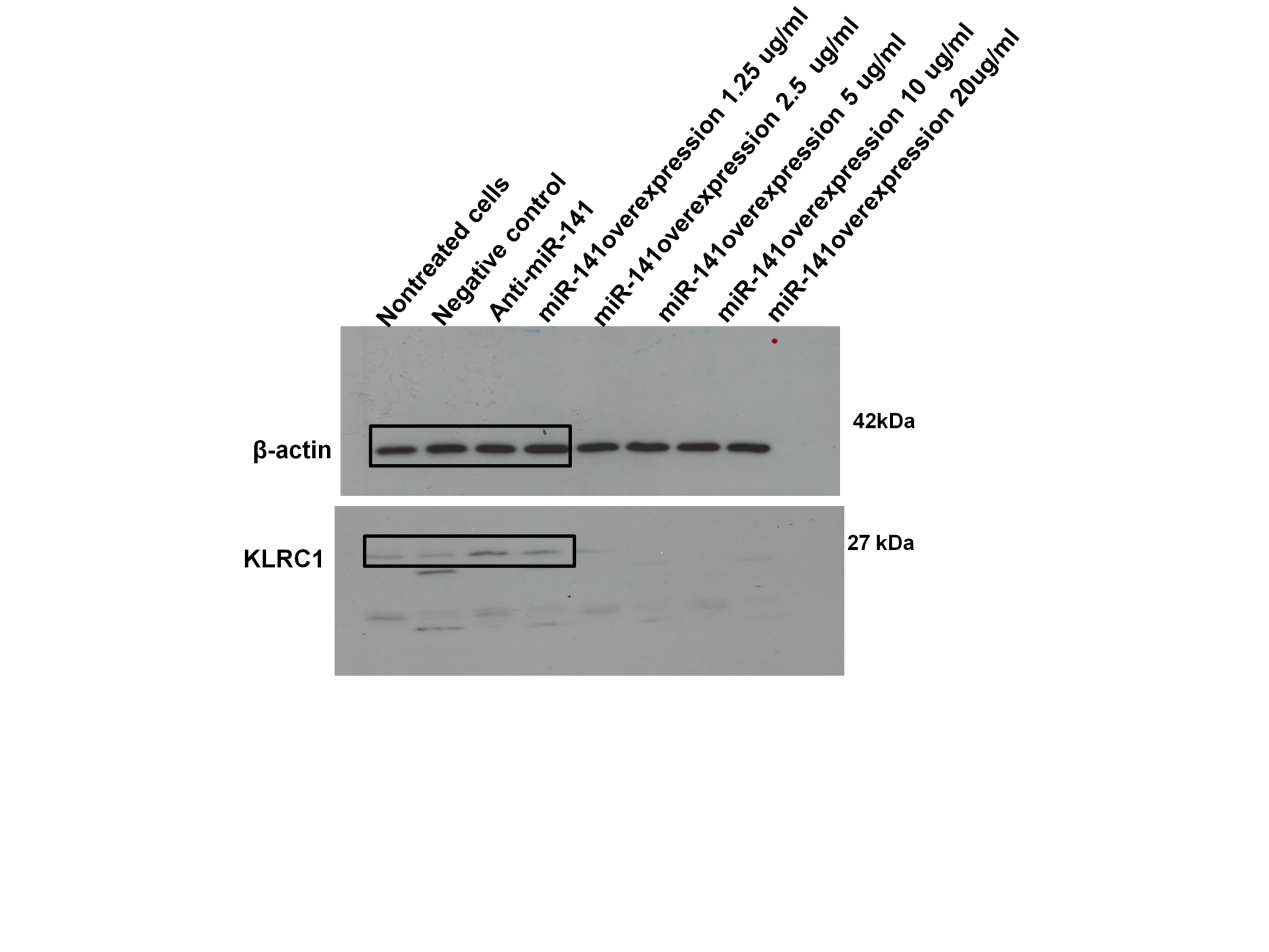
**

**Supp. Figure 1:**  Original immunoblotting membrane reveals KLRC1 protein expression and β-actin levels in HeLa cells that transfected with an inhibitor antagonist miR-141 or different concentrations of the miR-141 overexpression vector compared to nontransfected cells and control transfected cells. β-actin was introduced as an internal control.


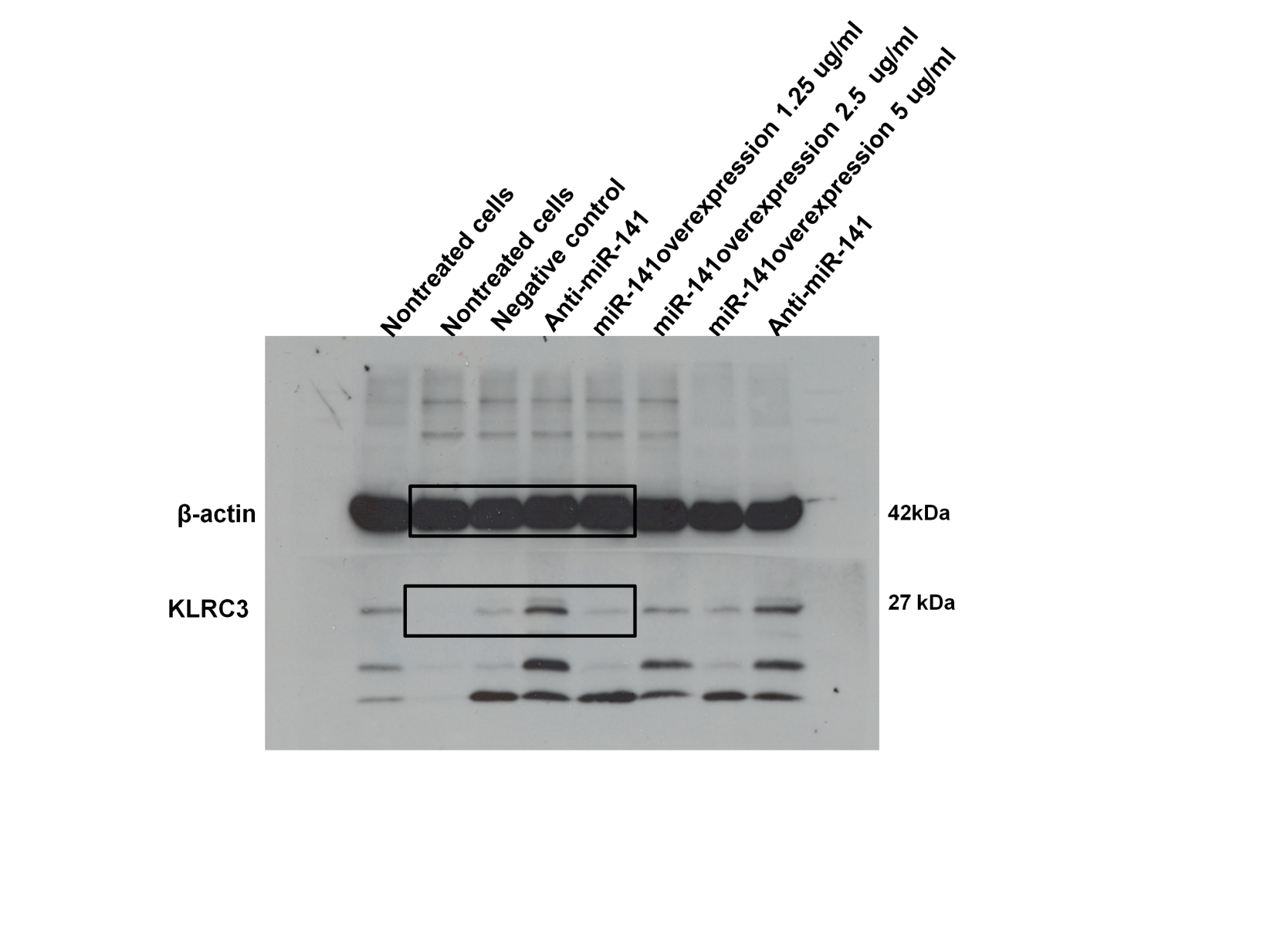


**Supp. Figure 2:**  Original immunoblotting membrane reveals KLRC3 protein expression and β-actin level in HeLa cells that transfected with an inhibitor antagonist miR-141 or different concentrations of the miR-141 overexpression vector compared to nontransfected cells and negative control transfected cells that treated with the same concentration of transfection reagent. β-actin was introduced as an internal control.


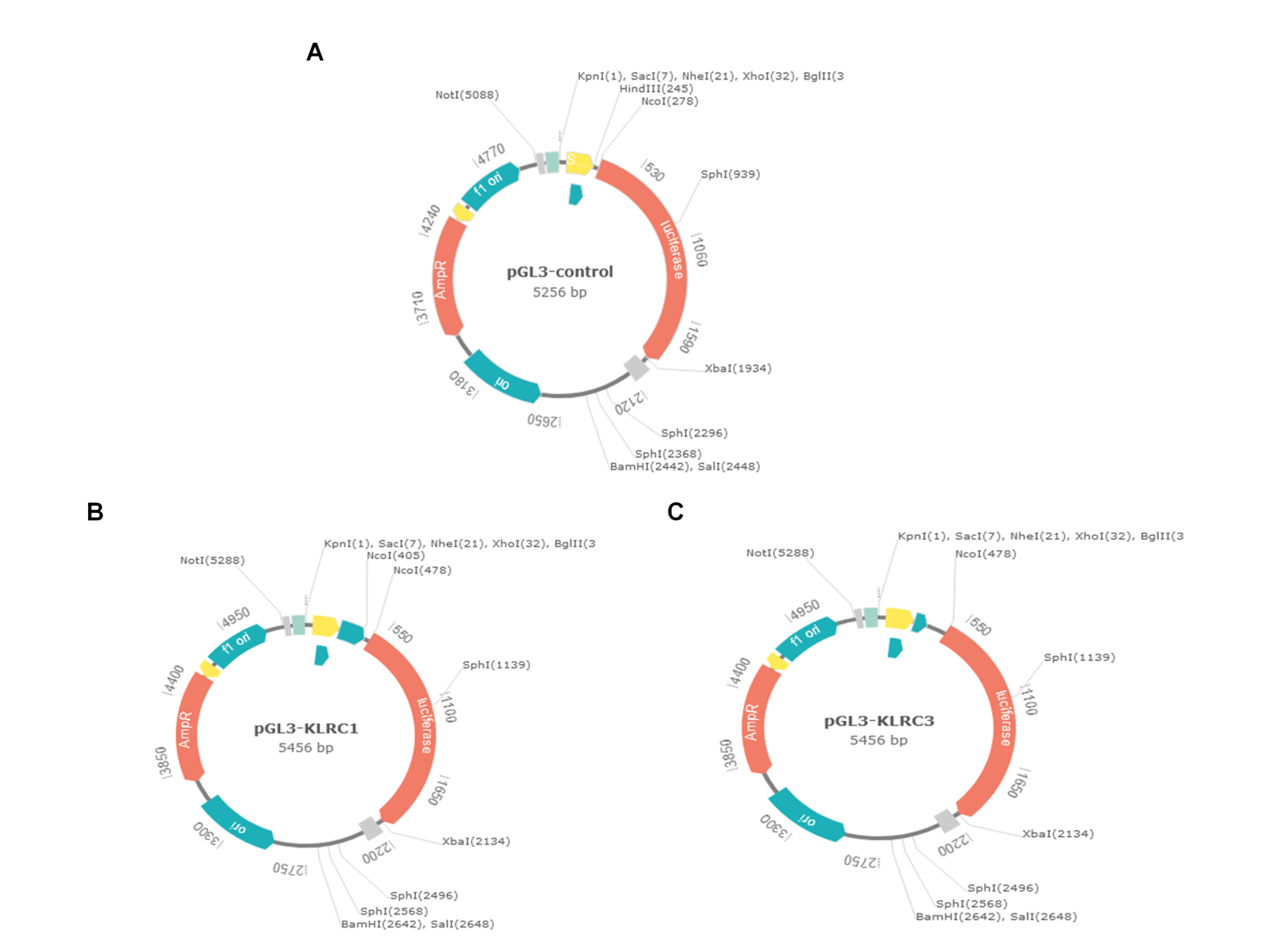


**Supp. Figure 3:** Schematic representation of luciferase reporter constructs map (A) pGL3-control vector, (B) pGL3-KLRC1 construct, and (C) pGL3-KLRC3 showed the cloned seeding region in the coding sequences of each targeted gene inserted between SV40 promoter and luciferase reporter gene using online Molbiotools.


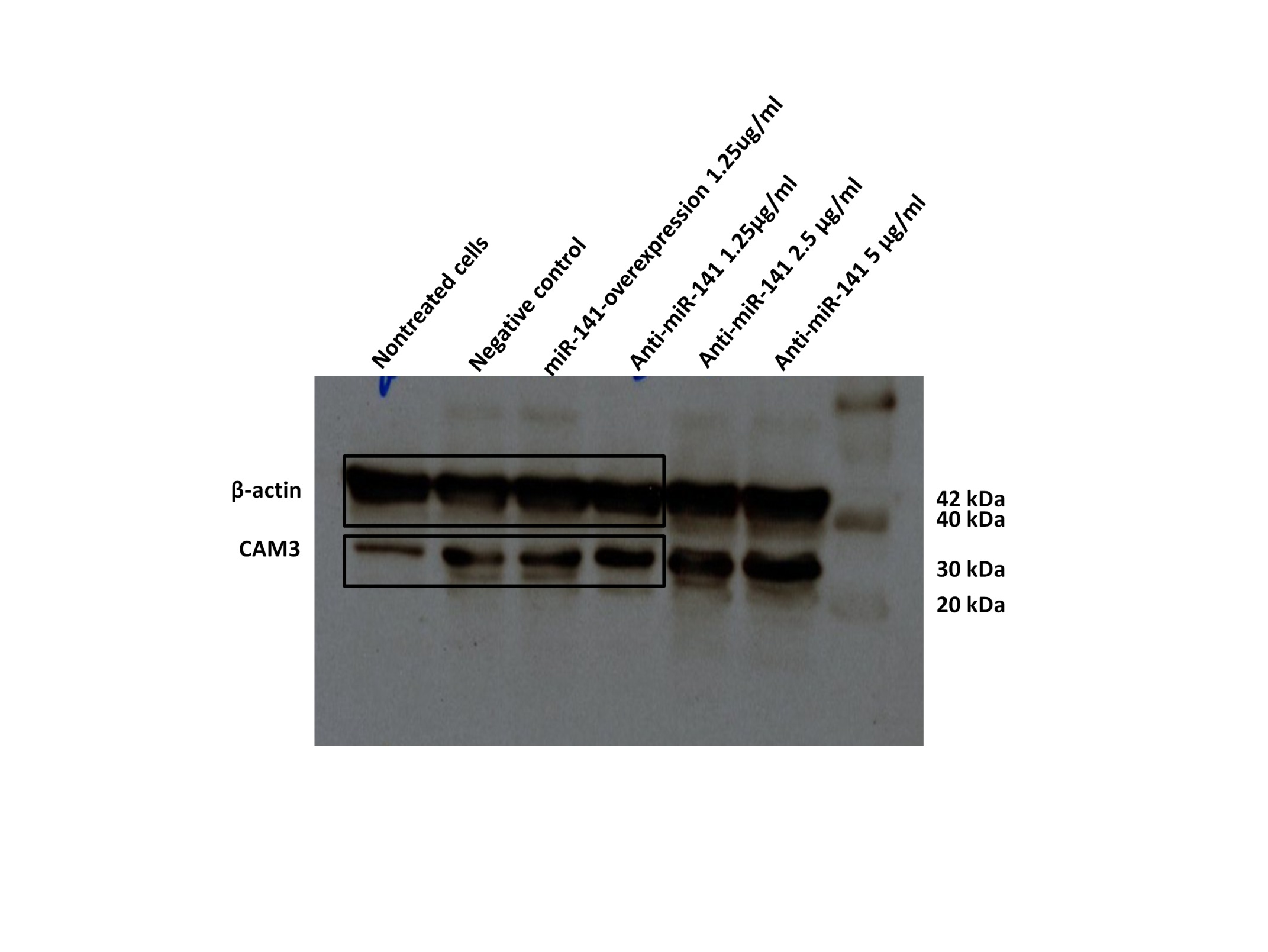


**Supp. Figure 4:** Original immunoblotting membrane reveals CAM 3 protein expression and β-actin levels in HeLa cells that transfected with an inhibitor antagonist miR-141 or the miR-141 overexpression vector compared to nontransfected cells and other control transfected cells. β-actin was introduced as an internal control. β-actin was introduced as an internal control.


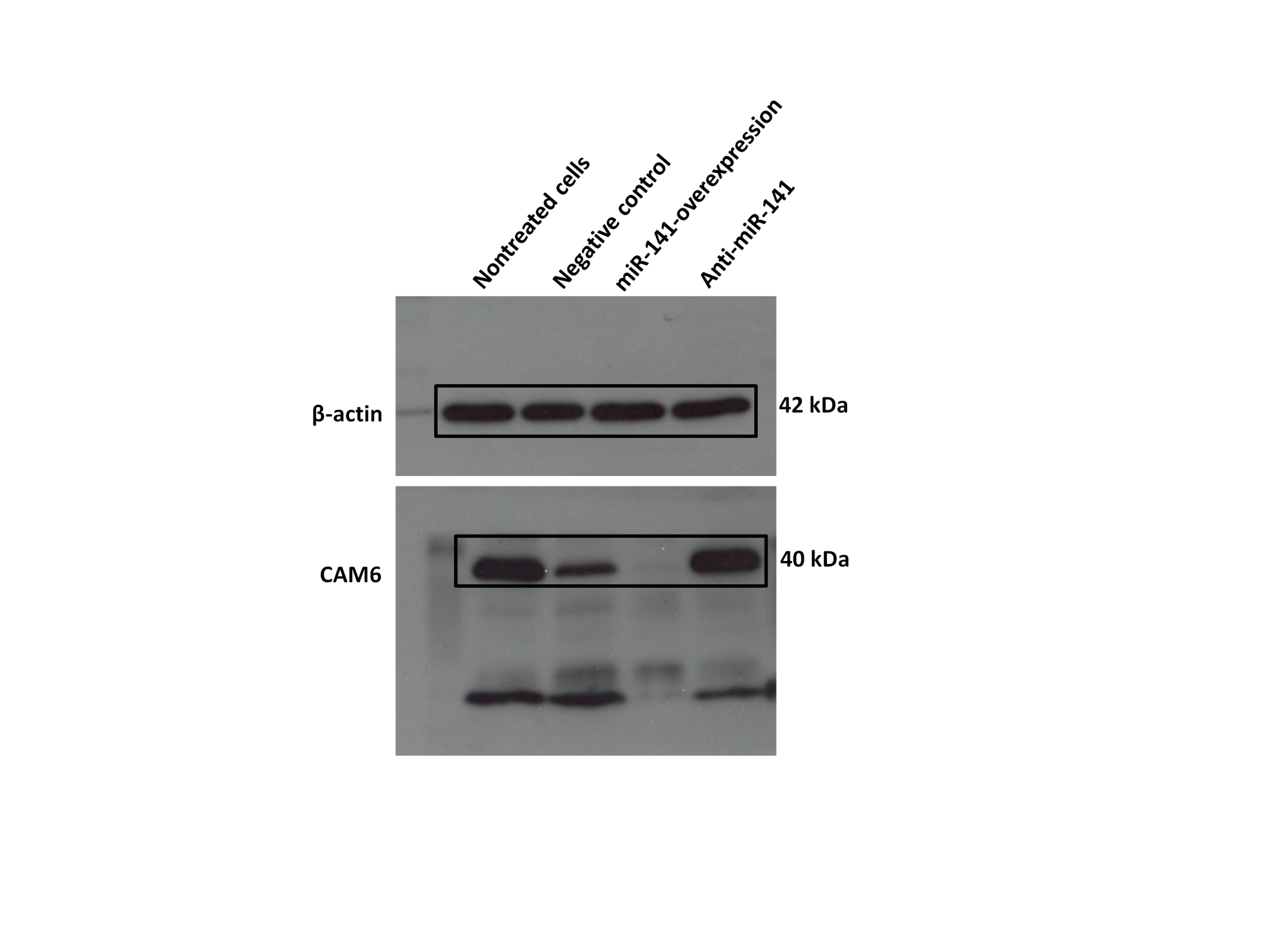


**Supp. Figure 5:** Original immunoblotting membrane reveals CAM 6 conjugated protein expression and β-actin levels in HeLa cells that transfected with an inhibitor antagonist miR-141 or different concentrations of the miR-141 overexpression vector compared to nontransfected cells and other control transfected cells. β-actin was introduced as an internal control. β-actin was introduced as an internal control.


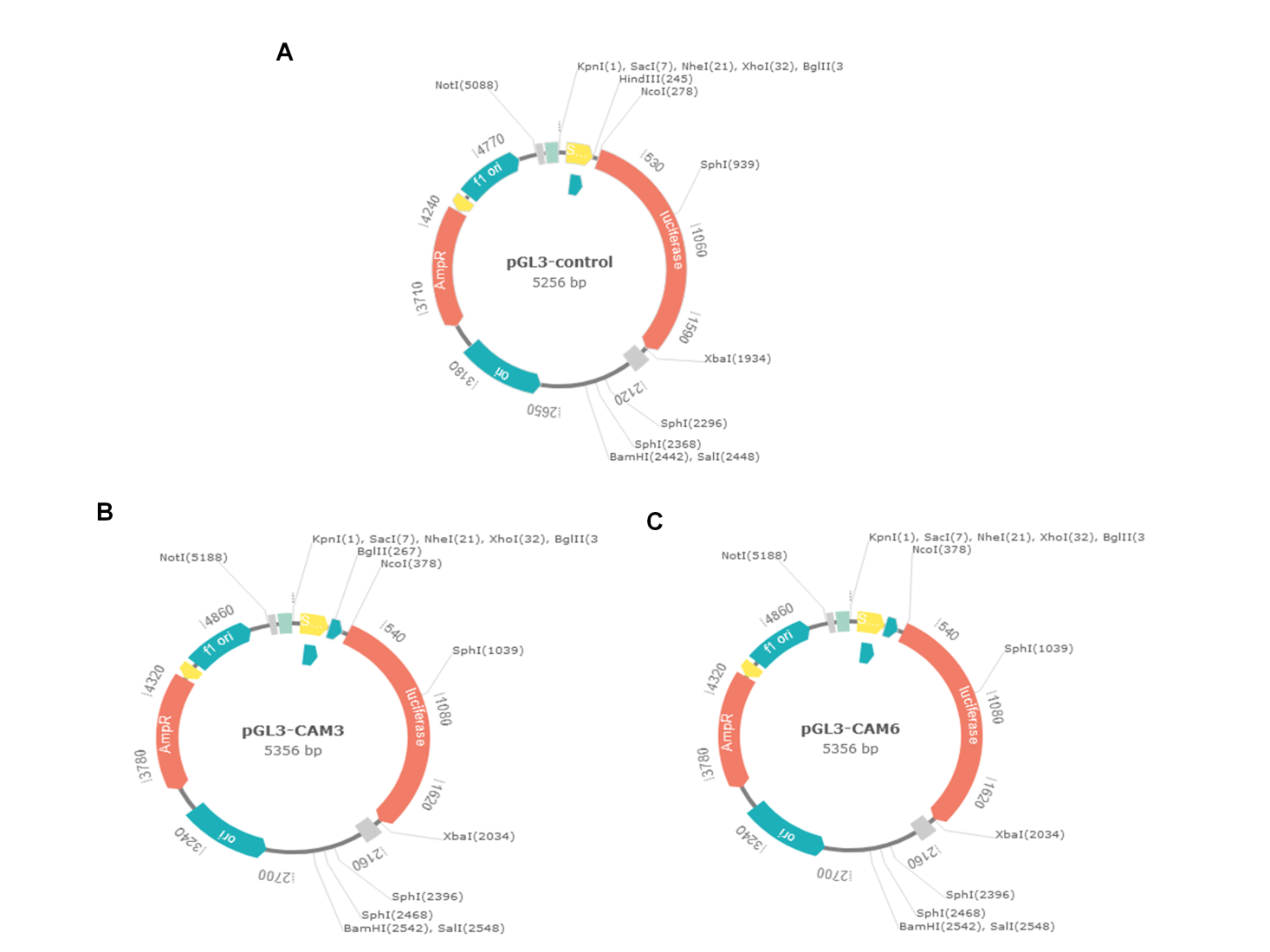


**Supp. Figure 6:** Schematic representation of luciferase reporter constructs map (A) pGL3-control vector, (B) pGL3-CAM3 construct, and (C) pGL3-CAM6 showed the cloned seeding region in the coding sequences of each targeted gene inserted between SV40 promoter and luciferase reporter gene using online Molbiotools.
